# In Vitro Anti-*Toxoplasma gondii* and Antimicrobial Activity of Amides Derived from Cinnamic Acid

**DOI:** 10.3390/molecules23040774

**Published:** 2018-03-28

**Authors:** Graziela Rangel Silveira, Karoline Azerêdo Campelo, Gleice Rangel Silveira Lima, Lais Pessanha Carvalho, Solange Silva Samarão, Olney Vieira-da-Motta, Leda Mathias, Carlos Roberto Ribeiro Matos, Ivo José Curcino Vieira, Edesio José Tenório de Melo, Edmilson José Maria

**Affiliations:** 1 Laboratório de Ciências Químicas, Centro de Ciências e Tecnologia, Universidade Estadual do Norte Fluminense-Darcy Ribeiro, Av. Alberto Lamego, 2000—Parque Califórnia, 28013-602 Campos dos Goytacazes/RJ, Brazil; karolcampelo16@yahoo.com.br (K.A.C.); gleice.sil@hotmail.com (G.R.S.L.); leddam8@gmail.com (L.M.); matos@uenf.br (C.R.R.M.); curcino@uenf.br (I.J.C.V.); edmilson_maria@yahoo.com.br (E.J.M.); 2 Laboratório de Biologia Celular e Tecidual, Centro de Biociências e Biotecnologia, Universidade Estadual do Norte Fluminense-Darcy Ribeiro, Av. Alberto Lamego, 2000—Parque Califórnia, 28013-602 Campos dos Goytacazes/RJ, Brazil; lais_pessanha@hotmail.com (L.P.C.); ejtm1202@gmail.com (E.J.T.M.); 3 Laboratório de Sanidade Animal, Centro de Ciências e Tecnologias Agropecuárias, Universidade Estadual do Norte Fluminense-Darcy Ribeiro, Av. Alberto Lamego, 2000—Parque Califórnia, 28013-602 Campos dos Goytacazes/RJ, Brazil; solangesamarao@gmail.com (S.S.S.); olney.motta@gmail.com (O.V.-d.-M.)

**Keywords:** synthetic amides, antiparasitic, antibiotic, acrylamides

## Abstract

Most cinnamic acids, their esters, amides, aldehydes, and alcohols present several therapeutic actions through anti-inflammatory, antitumor, and inhibitory activity against a great variety of microorganisms. In this work, eight amines derived from cinnamic acid were synthesized and tested against host cells infected with *Toxoplasma gondii* and the bacteria *Escherichia coli, Pseudomonas aeruginosa, Staphylococcus epidermidis,* and three strains of *Staphylococcus aureus*. Compounds **3** and **4** showed the best result against intracellular *T. gondii*, presenting antiparasitic activity at low concentrations (0.38 and 0.77 mM). The antibacterial activity of these compounds was also evaluated by the agar microdilution method, and amides 2 and 5 had a minimum inhibitory concentration of 250 µg mL^−1^ against two strains of *S. aureus* (ATCC 25923 and bovine strain LSA 88). These also showed synergistic action along with a variety of antibiotics, demonstrating that amines derived from cinnamic acid have potential as pharmacological agents.

## 1. Introduction

The cinnamic acids belong to a group of (C_6_-C_3_) substances derived from the secondary metabolism of plants. The term “cinnamic” refers to *Cinnamomum verum (Sin. C. zeylanicum)*, commonly known as the “cinnamon tree”. From this plant, “cinnamon” is obtained by drying the central part of the bark [1,2].

Most of the cinnamic acids, their esters, amides, aldehydes, and alcohols present several therapeutic actions like anti-inflammatory [3,4], antitumor [5], antimalarial [6,7], antitrypanosomal [8], and inhibitory activity against a broad range of microorganisms [2,9,10,11]. The mechanism of action is still poorly known [2]. Several researchers tried to produce alternative compounds obtained from derivates of this acid to potentiate its therapeutic properties.

Despite the broad range of biologic activities described in the derivates of the cinnamic acid, there has been no literature record until now on its effect against the protozoan parasite *Toxoplasma gondii*, the causative agent of toxoplasmosis [12,13]. One-third of the global population is estimated to be infected by this disease [12,14]. The most used treatment for this disease is the joint use of sulfadiazine and pyrimethamine [15]. This combination of drugs shall be used as a reference for other therapeutic protocols [13,15,16] to reduce the collateral effects associated with the treatment [16]. Several studies using thiosemicarbazones and thiazolidines aimed to reduce the toxic effects of the treatment [17,18,19].

Studies on the amides derived from cinnamic acid described inhibitory activity against several main bacteria such as *Escherichia coli* and *Staphylococcus* spp. [2]. The development of alternative antimicrobials against pathogenic bacteria is of great interest to the pharmaceutical industry [20].

**Figure 1:**
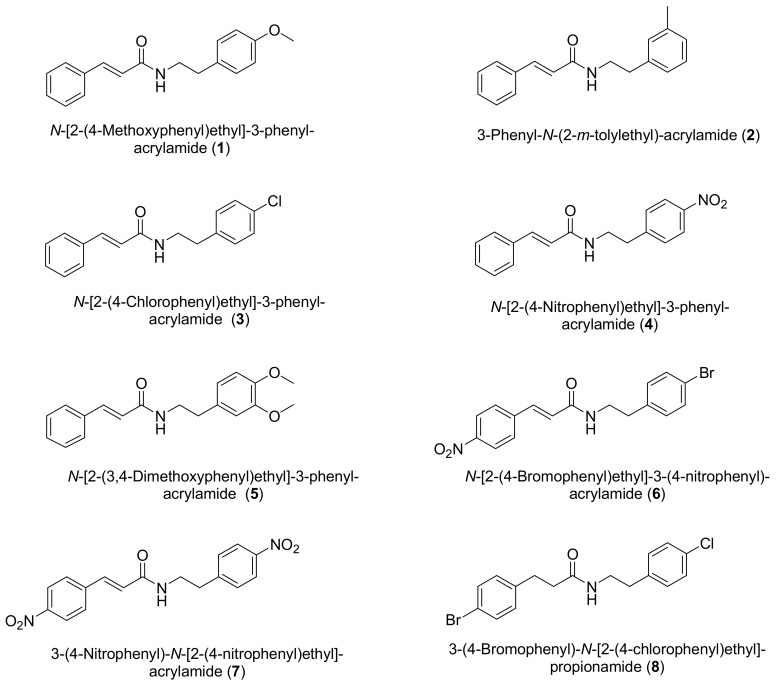
Chemical structure of compounds **1**–**8**.

With the aim of verifying the antimicrobial and antiparasitic activity, we synthesized eight amides derived from the cinnamic acid (Figure 1).

## 2. Results and Discussion

### 2.1. Chemistry

The amides were synthesized by two different methods: coupling of the derivates of the cinnamic acids with phenylethylamines substituted by dicyclohexylcarbodiimine (DCC) as a coupling agent (to obtain amides **1**–**5** and **8**) and nucleophilic substitution reaction using *p-*nitrophenylethylamine, *p-*bromophenylethylamine, and *trans*-*p-*nitrocynnamoil chloride (to obtain amines **6** and **7**). Among these reactions, those with acid chloride displayed higher yield.

The products of the reactions were solid at room temperature. These were analyzed using a nuclear magnetic resonance spectrometer. In all compounds, a quartet signal in the ^1^H-NMR spectrum was visible between 3.64–3.74 ppm, associated to the methylene adjacent to the nitrogen atom of the amide. In the ^13^C-NMR spectrum, signals relating to carbonyl groups of the amides were present between 164–172 ppm. The confirmation of these groups was also found in the HMBC spectrum by the correlation at two and three bonds between the carbonyl carbon and the adjacent hydrogens. The adsorptions in the infrared spectrum in the ranges of 1639–1657 and 3277–3347 cm^−1^ are characteristic of the C=O and N-H stretch, respectively. To verify the functional groups and to assess the purity of the obtained products and their molecular weight we used gas chromatography coupled to the mass spectrophotometer (GC-MS).

In this way, all the synthesized compounds showed spectral data consistent with the respective structures.

Of the eight synthesized compounds, five are unpublished as of yet (**2**, **4**, **6**–**8**).

### 2.2. Assay for Anti-Toxoplasma Gondii Activity

We focused the tests against *T. gondii* on the amides **1**, **3**–**7**. For this purpose, they were incubated for 24 h in cultures of LCC-MK2 cells infected and non-infected with *T. gondii*.

**Figure 2:**
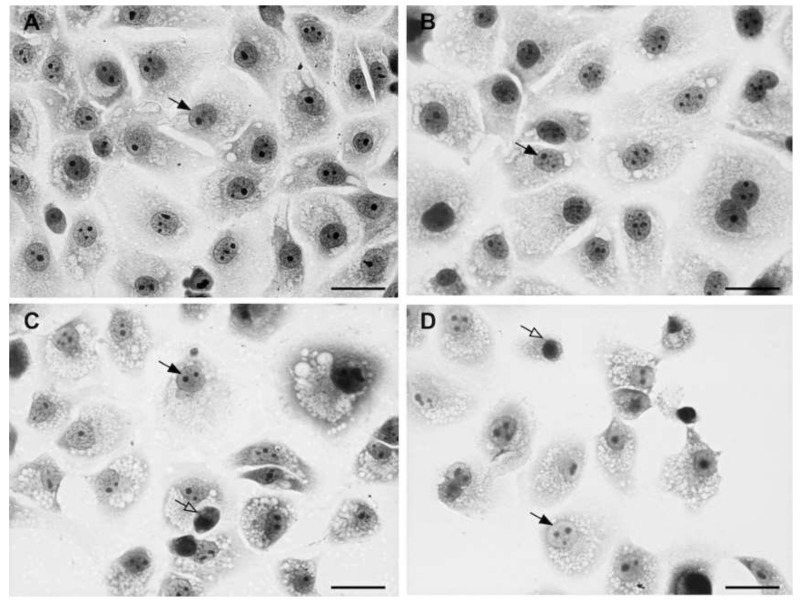
Toxicity in uninfected LLC-MK2 cells. The black arrow indicates nuclei with physiological appearance. White arrows indicate nuclear morphological changes indicative of toxicity. (**a**) Control case; In (**b**–**d**) a gradual increase of cytotoxicity is visible, associated with significant nuclear disorders and a decrease of the cellular number.

The cellular toxicity test was performed to assess the concentration of the synthesized amides causing neither toxic effects nor cell death in LCC-MK2 cells. The presence of morphological alterations, degenerations or cell death induced due to the treatment with the different compounds defined the presence of cellular toxicity. In general, increased cellular toxicity is directly proportional to the increase in concentration, as displayed in Figure 2. The figure highlights that, as compared to the control (Figure 2a), increasing the concentration (Figure 2b–d) caused nuclear morphological changes and the progressive increase of toxicity with the reduction of cell numbers.

Table 1 displays the results of the toxicity test in uninfected cells. Data show that compounds **6** and **7** exhibited no toxicity at 2.92 mM and are, therefore, the least toxic. Compound **5** had a similar behavior at 1.69 mM. Compounds **3** and **4** showed no toxicity at concentrations of 0.35 and 0.17 mM, respectively.

Compounds **1**, **3**, and **4** displayed higher cellular damages at the concentration of 2.92 mM. At this concentration, these compounds caused 100% of cellular death. No substance caused significant damages at levels equal or lower than 0.17 mM. The 1.69 mM concentration of compound 1 caused the reduction of 50% of the LLC-MK2 cells. The 2.92 mM concentration of compound 5 caused cellular damages in 69% of the cells.

**Figure 3:**
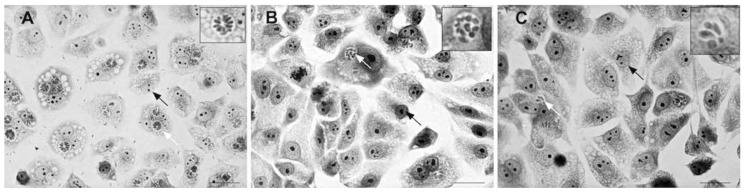
Optical microscopy of the biological test showing infected LLC-MK2 cells. The black arrows point to the cell nucleus; the white arrows indicate the parasitophorous vacuole. (**a**) Control case. Reduction of infection with the elimination of the parasite in the cells treated with (**b**) compound 1 and (**c**) **7**.

To define the dose of each compound which does not cause toxicity in uninfected cells, we tested cells infected with *T. gondii* at concentrations lower than the toxic dose established. After the infection of the LLC-MK2 cells with the parasite, we quantified the parasites based on the presence of tachyzoites. Infection of the *T. gondii* parasite was visible in untreated (control) cells. After treatment with the compounds, the number of infected host cells was reduced due to the elimination of the intracellular parasites (Figure 3).

Table 2 displays the effect of compounds **1**, **3**–**7** on LLC-MK2 cells and intracellular parasites. The number of cells in the control tests and the treatments are similar, suggesting no cytotoxicity of the tested compounds at the concentration used in the experiment.

During the incubation, tachyzoites displayed morphologic disorganization before being eliminated. These results corroborate with similar experiences using thiosemicarbazones and thiazolidines [17,18,19].

Compound **5** required a 0.16 mM concentration to be effective against the intracellular parasite. Compounds **3** and **4** eliminated 50% of the parasites at 0.077 and 0.38 mM, respectively. This concentration corresponds to 22.01 and 56.3 µg mL^−1^, which are the lowest effective doses against *T. gondii* among the compounds we tested. Compounds 6 and 7 showed better effects at concentrations of 1.33 and 1.46 mM, respectively (Table 2), although they had low cytotoxicity.

These results align with similar experiments performed by other researchers which studied the anti-toxoplasmic activity of thiosemicarbazone and thiazolidine analogues. Their results showed, the active concentrations of these drugs varied between 0.1 mM and 1.5 mM and cytotoxicity was between 0.5 and 10 mM [17,18,19]. Regarding the activity of the compounds used in this study, compounds **3** and **4** displayed better results at lower concentrations. This data suggests that the substitution of chlorine and nitro (NO_2_) in the *para* position in the ethylphenylamine portion promotes a significative action on the parasite.

The analysis of both cytotoxicity and antitoxoplasmic activity shows that the presence of an active electron scavenger group (NO_2_) in the *para* position of the cinnamoyl portion (as in compounds 6 and 7) are characteristic of the least cytotoxic compounds. Similarly, these compounds display the highest concentration for the induction of parasite mortality.

All the compounds had antiparasitic activity at concentrations lower than the standard drugs hydroxyurea (DL_50_ 4 mM) [18] and sulfadiazine (DL_50_ 3 mM) [19]. The high concentration of these drugs suggests the possibility of toxic effects.

### 2.3. Assay for Antimicrobial Activity

All synthesized compounds were tested for their antimicrobial activity by the broth microdilution method according to the Standards Institute and Laboratory [21]. Minimum inhibitory concentration (MIC) values were the lowest concentration that presented growth between 30 and 300 colonies.

Compounds **2** and **5** showed MICs of 250 µg mL^−1^ against strains of *Staphylococcus aureus* ATCC 25923 and LSA 88, respectively (Table 2). Most of the compounds displayed some activity against the tested strains at concentrations higher than 250 µg mL^−1^. Compounds **1**–**5** were effective against *Pseudomonas aeruginosa* ATCC 15442. Compound **2** was effective against *Staphylococcus aureus* LSA 88 (clinical strain isolated from bovine mastitis) and *Staphylococcus aureus* ATCC 33591. Compounds **6** and **7** were effective against *Escherichia coli* ATCC 25922; compounds **1**, **6** and **7** were effective against *Staphylococcus epidermidis* ATCC 12228^−1^. Compound **8** displayed no inhibitory activity on the tested microorganisms (Table 3).

Georgiev et al. [22] synthesized cinnamoylamides effective against *S. aureus* strains (62.5–500 µg mL^−1^) as observed in the present study.

Table 4 displays the antibiograms of the bacteria treated with the compounds **2** and **5**. The comparison between control bacteria and those treated with compound **2** after the exposition to the antibiotics displays larger inhibition halos of the strain ATCC 25923 (*p* < 0.01) for all the antibiotics that have been tested together with compound **2**. The increase of halo size of the compounds treated with compound **2** demonstrates that this product has synergic actions with these antibiotics, boosting their effects.

Compound **5** had a significant synergism (*p* < 0.05) on the effect of Sulfazotrim (SUT), Cefalexin (CFX), and Erythromycin (ERI) against the *S. aureus* bovine clinical sample LSA 88. On the contrary, the same compound **5** had an antagonistic effect toward AMO, reducing its inhibition halo (*p* < 0.05). Carlos et al. [20] also described the antagonistic effect between the control and the *Rauvolfia grandiflora* extracts with the antibiotics ampicillin, oxacillin, and cephalosporin.

Comparing the results among drugs, AMC and AMP displayed more significant activity in the control treatment for the ATCC 25923 strain. With the treatment of compound **2**, SUT activity is potentiated, producing a broader inhibition halo together with AMP. The LSA 88 strain displayed higher sensitivity toward penicillin G (PEN). This result remained after treatment with compound **5**.

The ATCC 25923 strain remained less sensitive to VAN and OXA even after the treatments, although an increase in the inhibition zone was confirmed (*p* < 0.01).

The results suggest the differences in the inhibition halos of *S. aureus* treated with compounds **2** and **5** increased the sensibility of strains ATCC 25923 and LSA 88 to different antibiotics.

## 3. Materials and Methods

### 3.1. General Information

All the chemical reagents used in this experiments were from Sigma Aldrich (St. Louis, MI, USA). Nuclear magnetic resonance spectra (NMR ^1^H, ^13^C, HMBC e HSQC) were obtained using BRUKER Avance III equipment (125 and 500 MHz) in deuterated chloroform (CDCl_3_) measured as parts per million (ppm) and coupling constants (*J*) in Hz. Infrared spectra were obtained using an IR-affinity spectrometer/Shimadzu (Tokyo, Japan) using KBr pellets. Purity and atomic mass of the compounds were confirmed by gas chromatography coupled with mass spectrometry (GC/MS), model 5975C Inert XL EI/CI/MS Agilent Technologies. All spectra are available in Supplementary Materials Figure S1–S48.

### 3.2. General Synthetic Procedure

The eight compounds were synthesized by the condensation reaction of carboxylic acids with phenylethylamines by activation of the acid portion; six compounds (**1**, **2**, **3**, **4**, **5** and **8**) were produced using *N*,*N*-dicyclohexylcarbodiimide (DCC-Sigma Aldrich, St. Louis, MI, USA) as the coupling agent. For compounds **6** and **7**, the corresponding acid chloride was used (Figure 1).

#### 3.2.1. General Synthetic Procedure for Compounds **1**–**5** and **8**

The addition of 0.2 mmol of cinnamic acid together with equimolar amounts of DCC and their amines in dichloromethane is done at 0 °C and stirred for 48 h under argon atmosphere. The reaction was monitored by thin layer chromatography and purified through a silica gel chromatographic column, eluting hexane and ethyl acetate (1:1) [9,24].

*N-[2-(4-Methoxyphenyl)ethyl]-3-phenyl-acrylamide* (**1**). Yellow amorphous solid; Yield: 39.9%; MP: 137.4–137.9 °C; IR (υ cm^−1^ KBr): υ 1624 (N-H), 3312 (N-H), 1655 cm^−1^ (C=O); NMR ^1^H (CDCl_3_, 500 MHz): *δ*_H_ 7.64 (*d*, *J* = 15.6 Hz, 1H, C=CH), 7.51–7.48 (*m*, 2H, Ar-H), 7.39–7.35 (*m*, 3H, Ar-H), 7.18–7.14 (*m*, 2H, Ar-H), 6.90–6.86 (*m*, 2H, Ar-H), 6.36 (*d*, *J* = 15.6 Hz, 1H, C=CH), 3.64 (*q*, *J* = 6.7 Hz, 2H, -CH_2_), 2.85 (*t*, *J* = 6.7 Hz, 2H, -CH_2_), 2.81 (*s*, 3H, OCH_3_); ^13^C-NMR (CDCl_3_, 125 MHz) *δ*_C_ 165.9 (C=O), 158.3 (C), 140.0 (CH), 134.8 (C), 130.8 (C), 129.8 (C), 129.7 (C), 128.8 (C), 127.8 (C), 120.7 (CH), 114.1 (C), 55.2 (OCH_3_), 41.0 (CH_2_), 34.8 (CH_2_); MS (EI), *m*/*z* = 281.0.

*3-Phenyl-N-(2-m-tolylethyl)-acrylamide* (**2**). Yellow amorphous solid; Yield: 49.4%; MP: 126.0–126.4 °C; IR (υ cm^−1^ KBr): υ 1618 (N-H), 3277 (N-H), 1657 (C=O) cm^−1^; ^1^H-NMR (CDCl_3_, 500 MHz): *δ*_H_ 7.64 (*d*, *J* = 15.3 Hz, 1H, C=CH), 7.52–7.48 (*m*, 2H, Ar-H), 7.40–7.35 (*m*, 3H, Ar-H), 7.07 (*m*, 1H, Ar-H), 7.10–7.03 (*m*, 3H, Ar-H), 6.36 (*d*, *J* = 15.3 Hz, 1H, C=CH), 3.68 (*q*, *J* = 6.8 Hz, 2H, -CH_2_), 2.88 (*t*, *J* = 6.8 Hz, 2H, -CH_2_), 2.38 (*s*, 3H, CH_3_); ^13^C-NMR (CDCl_3_, 125 MHz) *δ*_C_ 165.9 (C=O), 141.0 (CH), 138.9 (C), 138.5 (C), 134.9 (C), 129.7 (C), 129.7 (C), 128.8 (C), 128.6 (C), 127.8 (C), 127.3 (C), 125.8 (C), 120.7 (CH), 40.9 (CH_2_), 35.6 (CH_2_), 21.6 (CH_3_); MS (EI), *m*/*z* = 265.1.

*N-[2-(4-Chlorophenyl)ethyl]-3-phenyl-acrylamide* (**3**). White amorphous solid; Yield: 63.8%; MP: 163.4–164.0 °C; IR (υ cm^−1^ KBr): υ 1620 (N-H), 1651 (C=O) 3300 (N-H) cm^−1^; ^1^H-NMR (CDCl_3_, 500 MHz): *δ*_H_ 7.64 (*d*, *J* = 15.6 Hz, 1H, C=CH), 7.52–7.49 (*m*, 2H, Ar-H), 7.39–7.36 (*m*, 3H, Ar-H), 7.32–7.29 (*m*, 2H, Ar-H), 7.19–7.16 (*m*, 2H, Ar-H), 6.35 (*d*, *J* = 15.6 Hz, 1H, C=CH), 3.65 (*q*, *J* = 6.7 Hz, 2H, -CH_2_), 2.89 (*t*, *J* = 6.7 Hz, 2H, -CH_2_); ^13^C-NMR (CDCl_3_, 125 MHz) *δ*_C_ 165.9 (C=O), 141.3 (CH), 137.3 (C), 134.7 (C), 132.4 (C), 130.2 (C), 129.8 (C), 128.8 (C), 127.8 (C), 40.7 (CH_2_), 35.0 (CH_2_); MS (EI), *m*/*z* = 285.1.

*N-[2-(4-Nitrophenyl)ethyl]-3-phenyl-acrylamide* (**4**). Yellow amorphous solid; Yield: 41.2%; MP: 152.9–153.0 °C; IR (υ cm^−1^ KBr): υ 1612 (N-H), 1651 (C=O) 3312 (N-H) cm^−1^; ^1^H-NMR (CDCl_3_, 500 MHz): *δ*_H_ 8.20–8.15 (*m*, 2H, Ar-H), 7.66 (*d*, *J* = 15.3 Hz, 1H, C=CH), 7.52–7.49 (*m*, 2H, Ar-H), 7.42–7.36 (*m*, 5H, Ar-H), 6.39 (*d*, *J* = 15.3 Hz, 1H, C=CH), 3.71 (*q*, *J* = 7.0 Hz, 2H, -CH_2_), 3.04 (*t*, *J* = 7.0 Hz, 2H, -CH_2_); ^13^C-NMR (CDCl_3_, 125 MHz) *δ*_C_ 166.1 (C=O), 146.8 (C), 146.8 (C), 141.6 (CH), 134.6 (C), 129.9 (C), 128.9 (C), 127.8 (C), 123.9 (C), 120.2 (CH), 40.5 (CH_2_), 35.7 (CH_2_); MS (EI), *m*/*z* = 296.1. *N-[2-(3,4-Dimethoxyphenyl)ethyl]-3-phenyl-acrylamide* (**5**). Crystalline solid; Yield: 32.5%; MP: 110.8–111.0 °C; IR (υ cm^−1^ KBr): υ 1622 (N-H), 1655 (C=O) 3347 (N-H) cm^−1^; ^1^H RMN (CDCl_3_, 500 MHz): *δ*_H_ 7.64 (*d*, *J* = 15.6 Hz, 1H, C=CH), 7.52–7.47 (*m*, 2H, Ar-H), 7.39–7.34 (*m*, 3H, Ar-H), 6.80–6.74 (*m*, 2H, Ar-H), 6.84 (*m*, 1H, Ar-H), 6.36 (*d*, *J* = 15.6 Hz, 1H, C=CH), 3.88 (*s*, 6H, OCH_3_), 3.65 (*q*, *J* = 6.8 Hz, 2H, -CH_2_), 2.86 (*t*, *J* = 6.8 Hz, 2H, -CH_2_); ^13^C-NMR (CDCl_3_, 125 MHz) *δ*_C_ 165.9 (C=O), 149.1 (C), 147.8 (C), 141.1 (CH), 131.4 (C), 129.7 (C), 128.8 (C), 127.9 (C), 120.7 (C), 120.6 (CH), 112.0 (C), 111.4 (C), 56.0 (OCH_3_), 55.9 (OCH_3_), 41.0 (CH_2_), 35.2 (CH_2_); MS (EI), *m*/*z* = 311.1. *3-(4-Bromophenyl)-N-[2-(4-chlorophenyl)ethyl]-propionamide* (**8**). Crystalline solid; Yield: 64.0%; MP: 130.0–131.1 °C; IR (υ cm^−1^ KBr): υ 1639 (C=O), 3290 (N-H est) cm^−1^; ^1^H RMN (CDCl_3_, 500 MHz): δ 7.44–7.39 (*m*, 2H, Ar-H), 7.28–7.25 (*m*, 2H, Ar-H), 7.09–7.06 (*m*, 2H, Ar-H), 7.04–6.99 (*m*, 2H, Ar-H), 3.47 (*q*, *J* = 6.9 Hz, 2H, -CH_2_), 2.92 (*t*, *J* = 7.5 Hz, 2H, -CH_2_), 2.73 (*t*, *J* = 6.9 Hz, 2H, -CH_2_), 4.01 (*t*, *J* = 7.5 Hz, 2H, -CH_2_); ^13^C-NMR (CDCl_3_, 125 MHz): *δ*_C_ 171.6 (C=O), 139.8 (C), 137.2 (C), 132.4 (C), 131.6 (C), 130.2 (C), 130.0 (C), 128.1 (C), 120.1 (C), 40.5 (CH_2_), 38.2 (CH_2_), 35.0 (CH_2_), 31.0 (CH_2_); MS (EI), *m*/*z* = 367.0.

#### 3.2.2. Synthetic Procedure for Compounds **6** and **7**

The addition of 0.2 mmol of *trans*-*p*-nitrocinnamoyl chloride together with diisopropylamine with equimolar amounts of their phenylethylamines in dichloromethane [4,9] was done at 0 °C and stirred for 48 h under argon atmosphere. The reaction was monitored by thin layer chromatography and purified through a separation funnel (dichloromethane to water).

*N-[2-(4-Bromophenyl)ethyl]-3-(4-nitrophenyl)-acrylamide* (**6**). Yellow crystalline solid; Yield: 89.6%; MP: 146.0–147.0 °C; IR (υ cm^−1^ KBr): υ 1618 (N-H), 1651 (C=O) 3292 (N-H) cm^−1^; ^1^H-NMR (CDCl_3_, 500 MHz): *δ*_H_ 8.25–8.22 (*m*, 2H, Ar-H), 7.66–7.63 (*m*, 2H, Ar-H), 7.68 (*d*, *J* = 15.7 Hz, 1H, C=CH), 7.48–7.45 (*m*, 2H, Ar-H), 7.13–7.10 (*m*, 2H, Ar-H), 6.47 (*d*, *J* = 15.7 Hz, 1H, C=CH), 3.67 (*q*, *J* = 6.8 Hz, 2H, -CH_2_), 2.88 (*t*, *J* = 6.8 Hz, 2H, -CH_2_); ^13^C-NMR (CDCl_3_, 125 MHz) *δ*_C_ 164.9 (C=O), 148.2 (C), 141.2 (C), 138.7 (CH), 137.6 (C), 131.8 (C), 130.5 (C), 128.4 (C), 124.5 (CH), 124.2 (C), 40.8 (CH_2_), 35.0 (CH_2_); MS (EI), *m*/*z* = 374.0.

*3-(4-Nitrophenyl)-N-[2-(4-nitrophenyl)ethyl]-acrylamide* (**7**). Red amorphous solid; Yield: 82.3%; MP: 149.6–151.2 °C; IR (υ cm^−1^ KBr): υ 1620 (N-H), 1651 (C=O) 3294 (N-H) cm^−1^; ^1^H-NMR (CDCl_3_, 500 MHz): *δ*_H_ 8.26–8.18 (*m*, 4H, Ar-H), 7.70 (*d*, *J* = 15.4 Hz, 1H, C=CH), 7.67–7.59 (*m*, 2H, Ar-H), 7.44–7.40 (*m*, 2H, Ar-H), 6.48 (*d*, *J* = 15.4 Hz, 1H, C=CH), 3.74 (*q*, *J* = 7.0 Hz, 2H, -CH_2_), 3.06 (*t*, *J* = 7.0 Hz, 2H, -CH_2_); ^13^C-NMR (CDCl_3_, 125 MHz) *δ*_C_ 164.9 (C=O), 148.3 (C), 146.9 (C), 146.5 (C), 140.9 (C), 139.1 (CH), 129.7 (C), 128.4 (C), 124.2 (C), 123.9 (C), 123.9 (CH), 40.6 (CH_2_), 35.6 (CH_2_); MS (EI), *m*/*z* = 341.1.

### 3.3. Anti-Toxoplasma gondii Activity

Cells of LLC-MK2 (monkey kidney fibroblasts) were seeded on culture plates with 24 sumps with RPMJ 1640 (Sigma Aldrich, USA) supplemented with 5% fetal bovine serum for 24 h at 37 °C, as described by Liesen et al. [18]. These cultures were treated with different concentrations of selected amides (ranging from 0.19 to 2.92 mM) to verify cytotoxicity. The dose which did not show cellular toxicity was determined by observation of morphological alterations when compared to the control.

Subsequently, for the anti-*T. gondii* activity, test cells were infected with tachyzoites (1:5) and incubated at 37 °C for 24 h. After confirmation of infection, the cultures were treated with the cinnamic acid derivatives at different concentrations (Table 2) for 24 h.

After treatment, the cultures were washed with phosphate buffer solution (PBS), fixed using 4% paraformaldehyde, and stained with GIEMSA solution. Subsequently, cultures were analyzed by optical microscopy (AXIOPLAN/Zeis, Jena, Germany). The number of infected and uninfected cells, as well as intracellular parasites, were quantified, and quantitative analyses were performed to verify anti-toxoplasma activity.

The data presented were performed in triplicate.

### 3.4. Antimicrobial Activity

The biofilm forming bacterial strains of *S. aureus* (ATCC 33591), *E. coli* (ATCC 25922), *P. aeruginosa* (ATCC 15442) and those strains which did not form biofilm—*S. aureus* (ATCC 25923), bovine strain *S. aureus* (LSA88 SEC+/SED+/TSST-1+) [25], and *S. epidermidis* (ATCC 12228)—were obtained from the bacterial collection of the Animal Health Laboratory (Laboratório de Sanidade Animal—LSA, Universidade Estadual do Norte Fluminense – Darcy Ribeiro, Campos dos Goytacazes, Brazil).

The in vitro antibacterial activity of the amides was investigated using the broth dilution method. Mueller Hinton agar (Acumedia, Lansing, MI, USA) was used as the bacterial growth medium. Stock solutions of the compounds were prepared in dimethyl sulfoxide (DMSO—Sigma Aldrich, St. Louis, MI, USA) and serial dilutions. The maximum concentration tested was 250 µg mL^−1^.

Through the photometric reading (Densimat—bioMérrieux, Marcy-l’Étoile, France) the inoculum containing 1 × 10^8^ CFU mL^−1^ corresponding to 0.5 McFarland was obtained from the culture broth in the log phase of growth and diluted to 1 × 10^6^ CFU mL^−1^. Tests with gentamicin (control antibiotic) at 10 µg mL^−1^ concentration were conducted simultaneously.

Subsequently, a cell culture plate was used for MIC trials and solutions of serially diluted chemical compounds (0.1 mL) were seeded into each sample of each bacterial strain (0.1 mL). These were incubated in a bacteriological oven at 37 °C for 24 h. The MIC of each compound tested was defined as the lowest concentration that presented growth between 30 and 300 colonies.

After determining the MIC value of each compound, a synergy test was performed to verify the potentiation of the action of the conventional antibiotics together with the products for *S. aureus* strains (ATCC 25923 and LSA 88). For the antimicrobial activity pattern, the disc diffusion method was used on Mueller Hinton agar, according to the guidelines of the National Committee of Clinical Laboratory Standards [23]. The disks contained the following drugs: penicillin G (PEN, 10UI), oxacillin (OXA, 1 μg), amoxicillin (AMO, 10 μg), amoxicillin/clavulanic acid (AMC, 20/10 μg), ampicillin (AMP, 10 μg), cefalexin (CFX, 30 μg), sulfazotrim (SUT, 25 μg), clindamycin (CLI, 2 μg), erythromycin (ERI, 15 μg), gentamicin (GEN, 10 μg), tetracycline (VAN, 30 μg), and vancomycin (VAN, 30 μg) (Laborclin, Pinhais, Brazil).

The inhibition halos formed around each disc were measured with a digital caliper (Mitutoyo/CD-6′′CSX-B) and values submitted to Tukey’s *t*-test [26] were 1 and 5%. All trials were performed in triplicate.

## 4. Conclusions

Eight amides derived from cinnamic acid were synthesized and characterized based on their physical, analytical, and spectrometric data. All amides tested showed anti-*T. gondii* activity, reducing the number of infected cells and intracellular parasites. Compound **3** and **4** displayed the best results. Compounds **1**, **2**, **3**, **4**, **5**, **6**, and **7** showed weak activity against different strains of bacteria at a concentration higher than 250 µg mL^−1^. Compounds **2** and **5** demonstrated activity against two strains of *S. aureus* (ATCC 25923 and LSA 88) at a concentration of 250 µg mL^−1^. The same compounds showed a synergic effect with different antibiotics. These results indicate that these classes of analogs are potential pharmacological agents.

## Figures and Tables

**Table 1:** Cytotoxicity of the compounds **1** and **3**–**7** in LLC-MK2 uninfected cells.

Cytotoxicity (Uninfected Culture) ^a^					
		Concentration (mM)			
**Drug**	Untreated	0.17	0.35	1.69	2.92
**Compound 1**	226 ± 09	-	229 ± 13	112 ± 14	0
**Compound 3**	196 ± 12	-	194 ± 11	-	0
**Compound 4**	196 ± 12	200 ± 21	-	0	0
**Compound 5**	226 ± 09	-	-	217 ± 30	71 ± 10
**Compound 6**	206 ± 10	-	-	-	209 ± 12
**Compound 7**	206 ± 10	-	-	-	209 ± 17

^a^ Values means ± SD.

**Table 2:** Effect of compounds **1** and **3**–**7** on cultures of LLC-MK2 cells infected and multiplication of *T. gondii*.

		Cytotoxicity (Infected Culture) ^a^			
Drugs	Concentration (mM)	Uninfected Cells	Infected Cells	Total of Cells	Parasites
**Control**	0	83 ± 8	28 ± 4	111 ± 12	179 ± 29
**Compound 1**	0.18	129 ± 6	24 ± 4	153 ± 10	166 ± 15
**Control**	0	108 ± 19	51 ± 6	159 ± 25	254 ± 38
**Compound 3**	0.077	147 ± 3	23 ± 5	170 ± 8	127 ± 32
**Control**	0	108 ± 19	51 ± 6	159 ± 25	254 ± 38
**Compound 4**	0.038	149 ± 24	35 ± 10	184 ± 34	134 ± 32
**Control**	0	83 ± 8	28 ± 4	111 ± 12	179 ± 29
**Compound 5**	0.16	114 ± 3	12 ± 1	126 ± 4	82 ± 19
**Control**	0	79 ± 7	37 ± 3	116 ± 10	174 ± 16
**Compound 6**	1.33	89 ± 12	31 ± 5	120 ± 17	83 ± 23
**Control**	0	79 ± 7	37 ± 3	116 ± 10	174 ± 16
**Compound 7**	1.46	104 ± 9	28 ± 3	132 ± 12	98 ± 12

^a^ Values means ± SD.

**Table 3:** Minimum inhibitory concentration (MIC) of compounds **1**–**8** against different bacterial strains.

Compound	Minimum Inhibitory Concentration (MIC) in µg mL^−1 a^					
	ATCC 15442	LSA 88	ATCC 33591	ATCC 25923	ATCC 25922	ATCC 12228
**1**	>250	−	−	−	−	−
**2**	>250	>250	>250	250	−	>250
**3**	>250	−	−	−	−	−
**4**	>250	−	−	−	−	−
**5**	>250	250	−	−	−	−
**6**	−	−	−	−	>250	>250
**7**	−	−	−	−	>250	>250
**8**	−	−	−	−	−	−

^a^ The maximum concentration tested was 250 µg mL^−1^. Gentamicin was used as control antibiotic (concentration 10 µg mL^−1^). Tests were performed in triplicate. - No inhibition at the concentration tested. >250 indicates that there was sensitivity, but the concentration should be higher than 250 µg mL^−1^ for a count of fewer than 300 colonies.

**Table 4:** Antibiogram (average halo diameter expressed in mm) of strains of *S. aureus* treated with amides derived from cinnamic acid and submitted to 12 different antibiotics. Assessed by agar gel diffusion method [23].

	Strains			
	ATCC 25923		LSA 88	
Antibiotic	Control	2	Control	5
**VAN**	16.74Bg	23.98Ad	17.50Ae	17.45Ai
**AMP**	27.05Ba	36.99Aa	33.17Aab	34.64Ab
**SUT**	25.00Bbc	37.00Aa	28.05Bc	29.68Ade
**CLI**	21.40Be	34.42Aab	26.76Ac	27.17Ag
**CFX**	23.70Bcd	34.86Aab	28.09Bc	28.90Aef
**ERI**	20.87Be	32.42Aabc	25.73Bc	27.99Afg
**AMO**	24.95Bbc	32.88Aab	33.98Aab	31.36Bc
**PEN**	25.69Bab	33.40Aab	36.11Aa	36.21Aa
**GEN**	21.37Be	30.24Abc	26.72Ac	26.99Ag
**AMC**	26.81Ba	35.52Aab	35.06Aab	35.62Aab
**OXA**	19.02Bf	26.61Acd	21.31Ad	21.32Ah
**TET**	22.40Bde	33.33Aab	32.49Ab	30.91Acd

The averages followed by the same small letter in the column (antibiotics) and a capital letter on the line (treatment with amines derived from cinnamic acid) do not differ from each other. Tukey test, 1 and 5% probability. VAN: Vancomycin; AMP: Ampicillin; SUT: Sulfazotrim; CLI: Clindamycin; CFX: Cefalexin; ERI: Erythromycin; AMO: Amoxicillin; PEN: Penicillin G; GEN: Gentamicin; AMC: Amoxicillin/ Clavulanic acid; OXA: Oxacillin; TET: Tetracycline.

## Supplementary Materials

MS and IV spectra, ^1^H-, ^13^C, HMBC, HSQC, and HMBC spectral data are available as supplementary materials online.

## References

[1] Pittman B.S. (2010). Cinnamon: It’s Not Just For Making Cinnamon Rolls. Ethnobot. Leafl..

[2] Guzman J.D. (2014). Natural Cinnamic Acids, Synthetic Derivatives and Hybrids with Antimicrobial Activity. Molecules.

[3] Boudreau L.H., Maillet J., LeBlanc L.M., Jean-François J., Touaibia M., Flamand N., Surette M.E. (2012). Caffeic acid phenethyl ester and its amide analogue are potent inhibitors of leukotriene biosynthesis in human polymorphonuclear leukocytes. PLoS ONE.

[4] Chan H.H., Hwang T.L., Thang T., Leu Y.L., Kuo P.C., Nguyet B.M., Dai D.N., Wu T.S. (2013). Isolation and Synthesis of Melodamide A, a New Anti-inflammatory Phenolic Amide from the Leaves of *Melodorum fruticosum*. Planta Med..

[5] Shi Z.-H., Li N.-G., Shi Q.-P., Tang H., Tang Y.-P., Li W., Yin L., Yang J.-P., Duan J.-A. (2013). Synthesis and structure-activity relationship analysis of caffeic acid amides as selective matrix metalloproteinase inhibitors. Bioorg. Med. Chem. Lett..

[6] Ferraz R., Pinheiro M., Gomes A., Teixeira C., Prudêncio C., Reis S., Gomes P. (2017). Effects of novel triple-stage antimalarial ionic liquids on lipid membrane models. Bioorg. Med. Chem. Lett..

[7] Wiesner J., Mitsch A., Wissner P., Jomaa H., Schlitzer M. (2001). Structure-activity relationships of novel anti-malarial agents. Part 2: Cinnamic acid derivatives. Bioorg. Med. Chem. Lett..

[8] Carvalho S.A., Feitosa L.O., Soares M., Costa T.E.M.M., Henriques M.G., Salomão K., De Castro S.L., Kaiser M., Brun R., Wardell J.L. (2012). Design and synthesis of new (E)-cinnamic *N*-acylhydrazones as potent antitrypanosomal agents. Eur. J. Med. Chem..

[9] Dai L., Zang C., Tian S., Liu W., Tan S., Cai Z., Ni T., An M., Li R., Gao Y. (2015). Design, synthesis, and evaluation of caffeic acid amides as synergists to sensitize fluconazole-resistant *Candida albicans* to fluconazole. Bioorg. Med. Chem. Lett..

[10] Montes R.C., Perez A.L., Medeiros C.I.S., Araújo M.O., Lima E.D., Scotti M.T., Sousa D.P. (2016). Synthesis, Antifungal Evaluation and In Silico Study of *N*-(4-Halobenzyl)amides. Molecules.

[11] Narasimhan B., Belsare D., Pharande D., Mourya V., Dhake A. (2004). Esters, amides and substituted derivatives of cinnamic acid: Synthesis, antimicrobial activity and QSAR investigations. Eur. J. Med. Chem..

[12] William J., Sullivan V.J. (2013). Mechanisms of Toxoplasma gondii persistence and latency. NIH Public Access.

[13] Tenter A.M., Heckeroth A.R., Weiss L.M. (2000). Toxoplasma gondii: From animals to humans. Int. J. Parasitol..

[14] Galván-Ramírez M.D.L., Gutiérrez-Maldonado A.F., Verduzco-Grijalva F., Marcela J., Jiménez D. (2014). The role of hormones on Toxoplasma gondii infection: A systematic review. Front. Microbiol..

[15] Galvani A.T. (2014). Toxoplasma gondii: Um novo desafio. Núcleo Pesquisas em Avaliação Riscos Ambientais.

[16] Wei H., Wei S., Lindsay D.S., Peng H. (2015). A Systematic Review and Meta-Analysis of the Efficacy of Anti-Toxoplasma gondii Medicines in Humans. PLoS ONE.

[17] Tenório R.P., Carvalho C.S., Pessanha C.S., Lima J.G., Faria A.R., Alves A.J., Melo E.J.T., Góes A.J.S. (2005). Synthesis of thiosemicarbazone and 4-thiazolidinone derivatives and their in vitro anti-Toxoplasma gondii activity. Bioorg. Med. Chem. Lett..

[18] Liesen A.P., Aquino T.M., Carvalho C.S., Lima V.T., Araújo J.M., Lima J.G., Faria A.R., Melo E.J.T., Alves A.J., Alves E.W. (2010). Synthesis and evaluation of anti-Toxoplasma gondii and antimicrobial activities of thiosemicarbazides, 4-thiazolidinones and 1,3,4-thiadiazoles. Eur. J. Med. Chem..

[19] Aquino T.M., Liesen A.P., Silva R.E.A., Lima V.T., Carvalho C.S., Faria A.R., Araujo J.M., Lima J.G., Alves A.J., Melo E.J.T. (2008). Synthesis, anti-Toxoplasma gondii and antimicrobial activities of benzaldehyde 4-phenyl-3-thiosemicarbazones 5-thiazolidineacetic acids. Bioorg. Med. Chem..

[20] De Almeida Carlos L., da Silva Amaral K.A., Curcino Vieira I.J., Mathias L., Braz-Filho R., Silva Samarão S., Vieira-da-Motta O. (2010). Rauvolfia Grandiflora (Apocynaceae) Extract Interferes with Staphylococcal Density, Enterotoxin Production and Antimicrobial Activity. Braz. J. Microbiol..

[21] Jorgensen J.H., Murray P.R., Baro E.J., Pfaller M.A., Tonover F.C. (1995). Manual of Clinical Microbiology.

[22] Georgiev L., Chochkova M., Ivanova G., Najdenski H., Ninova M., Milkova T. (2012). Radical scavenging and antimicrobial activities of cinnamoyl amides of biogenic monoamines. Riv. Ital. Sost. Grasse.

[23] CLSI (2015). Performance Standards for Antimicrobial Disk Susceptibility Tests; Approved Standard.

[24] Sheikh M.C., Takagi S., Yoshimura T., Morita H. (2010). Mechanistic studies of DCC/HOBt-mediated reaction of 3-phenylpropionic acid with benzyl alcohol and studies on the reactivities of “active ester” and the related derivatives with nucleophiles. Tetrahedron.

[25] Vieira-Da-Motta O., Folly M.M., Sakyiama C.C.H. (2001). Detection of different Staphylococcus aureus strains in bovine milk from subclinical mastitis using PCR and routine techniques. Braz. J. Microbiol..

[26] Silva F.A.S., Azevedo C.A.V. (2016). The Assistat Software Version 7.7 and its use in the analysis of experimental data.. Afr. J. Agric. Res..

