# Supplementary material for: In Vitro Anti-Toxoplasma gondii and Antimicrobial Activity of Amides Derived from Cinnamic Acid
Source: Molecules. 2018 Mar 28;23(4):774. doi: 10.3390/molecules23040774 (PMC6017938; doi:10.3390/molecules23040774)
Supplement: Supplementary file 1 [file molecules-23-00774-s001.pdf]

*molecules*

*Supplementary Materials*

# In vitro Anti-*Toxoplasma gondii* and Antimicrobial Activity of Amides Derivated From Cinnamic Acid

**Graziela Rangel Silveira[1,*], Karoline Azerêdo Campelo[1], Gleice Rangel Silveira Lima[1], Lais Pessanha Carvalho[2], Solange Silva Samarão[3], Olney Vieira-da-Motta[3], Leda Mathias[1], Carlos Roberto Ribeiro Matos[1], Ivo José Curcino Vieira[1], Edesio José Tenório de Melo[2], Edmilson José Maria[1]**

[1] Laboratório de Ciências Químicas, Centro de Ciências e Tecnologia, Universidade Estadual do Norte Fluminense-Darcy Ribeiro, Av. Alberto Lamego, 2000 - Parque Califórnia, Campos dos Goytacazes/RJ, 28013-602, Brasil; graziela_sil@hotmail.com (G. R. S.); karolcampelo16@yahoo.com.br (K. A. C.); gleice.sil@hotmail.com (G. R. S. L.); leddam8@gmail.com (L. M.); matos@uenf.br (C. R. R. M.); curcino@uenf.br (I. J. C. V.); edmilson_maria@yahoo.com.br (E. J. M.).

[2] Laboratório de Biologia Celular e Tecidual, Centro de Biociências e Biotecnologia, Universidade Estadual do Norte Fluminense-Darcy Ribeiro, Av. Alberto Lamego, 2000 - Parque Califórnia, Campos dos Goytacazes/RJ, 28013-602, Brasil; lais_pessanha@hotmail.com (L. P. C.); ejtm1202@gmail.com (E. J. T. M.).

[3] Laboratório de Sanidade Animal, Centro de Ciências e Tecnologias Agropecuárias, Universidade Estadual do Norte Fluminense-Darcy Ribeiro, Av. Alberto Lamego, 2000 - Parque Califórnia, Campos dos Goytacazes/RJ, 28013-602, Brasil; solangesamarao@gmail.com (S. S. S.); olney.motta@gmail.com (O. V.dM.).

* Corresponding author: graziela_sil@hotmail.com; Tel.: +55-022-2728-6167

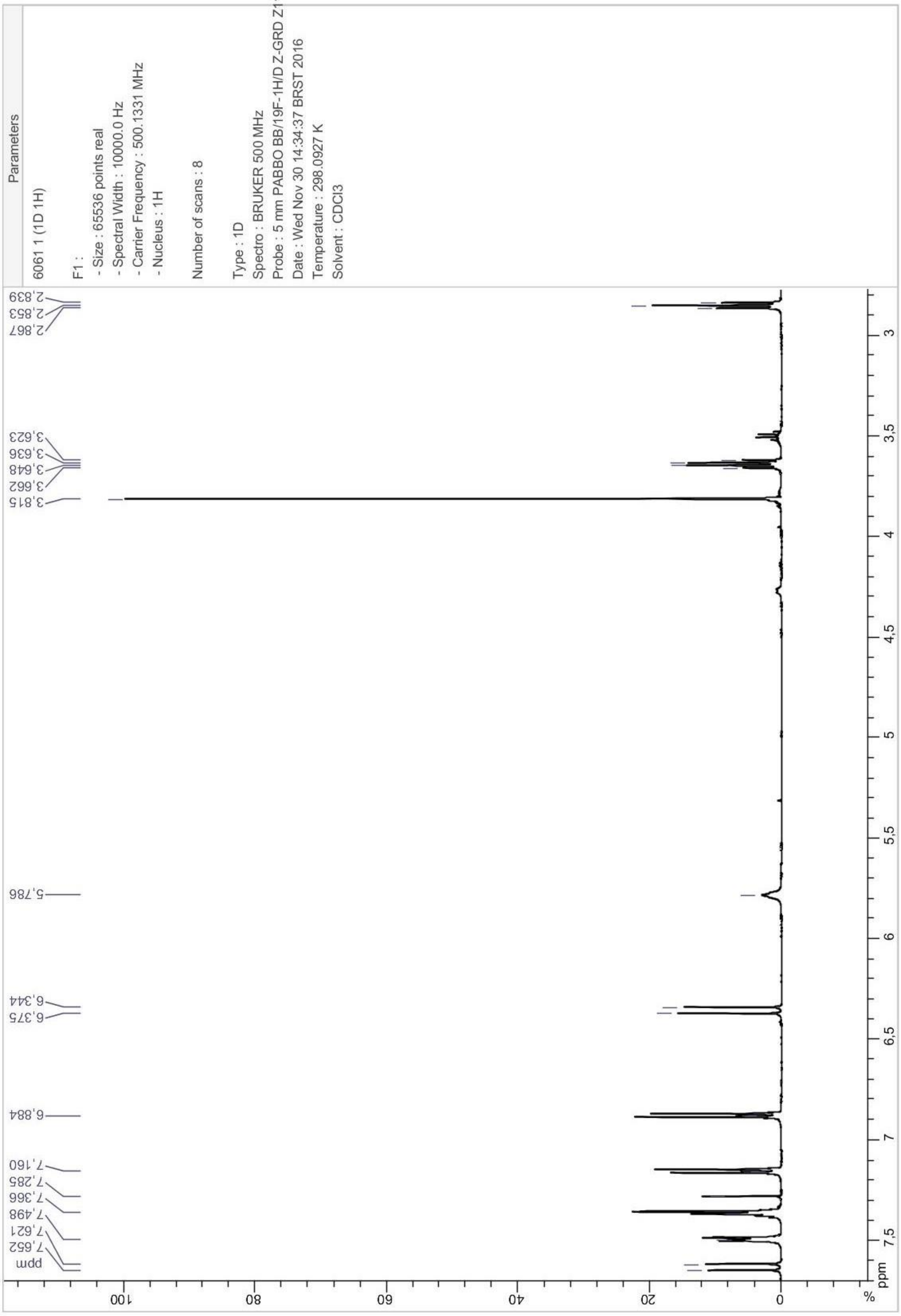


**Figure S1.** $^{1}H$ NMR spectrum of compound **1** ($CDCl_3$, 500 MHz).

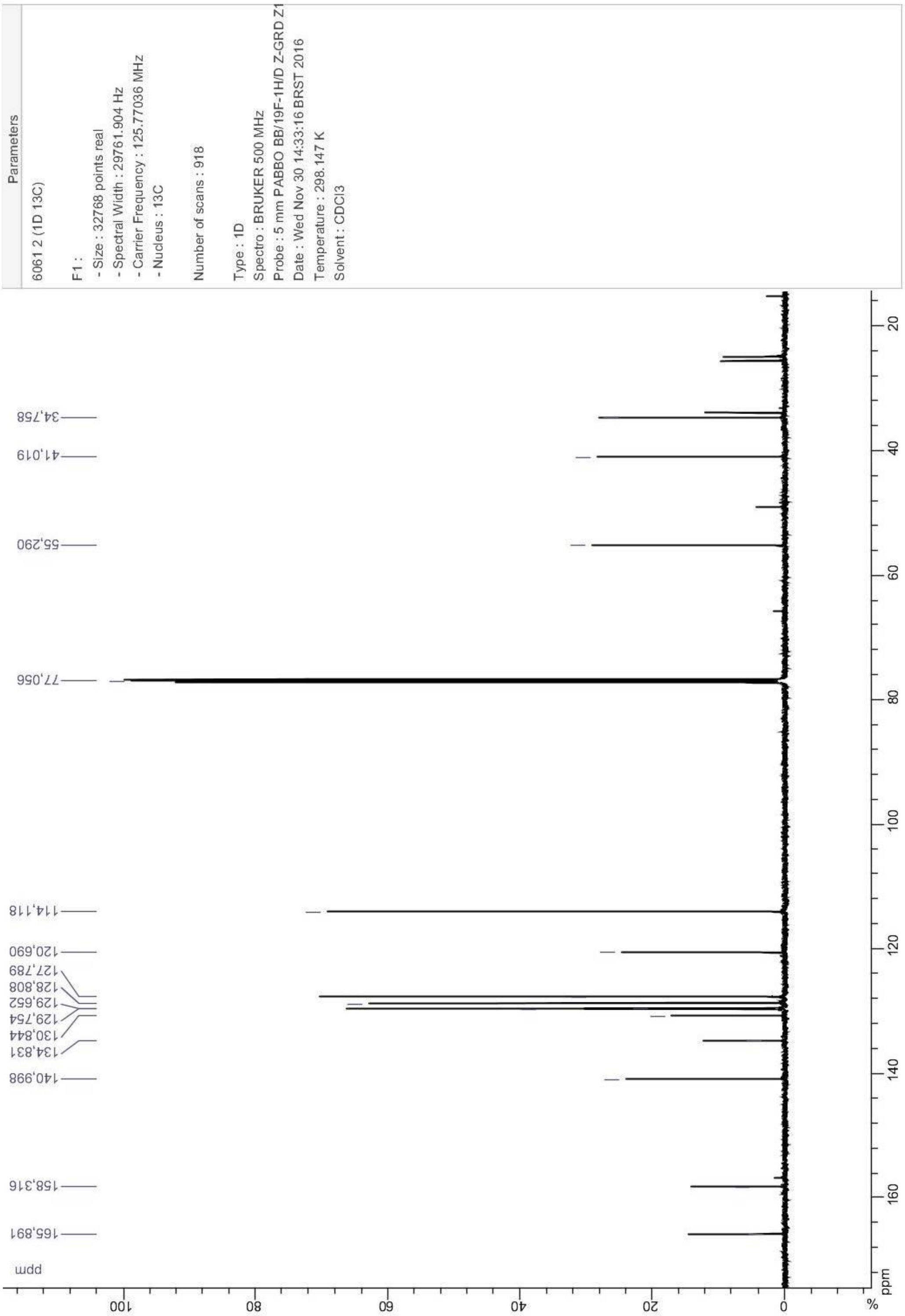


**Figure S2.** $^{13}C$ NMR spectrum of compound **1** ($CDCl_3$, 125 MHz).

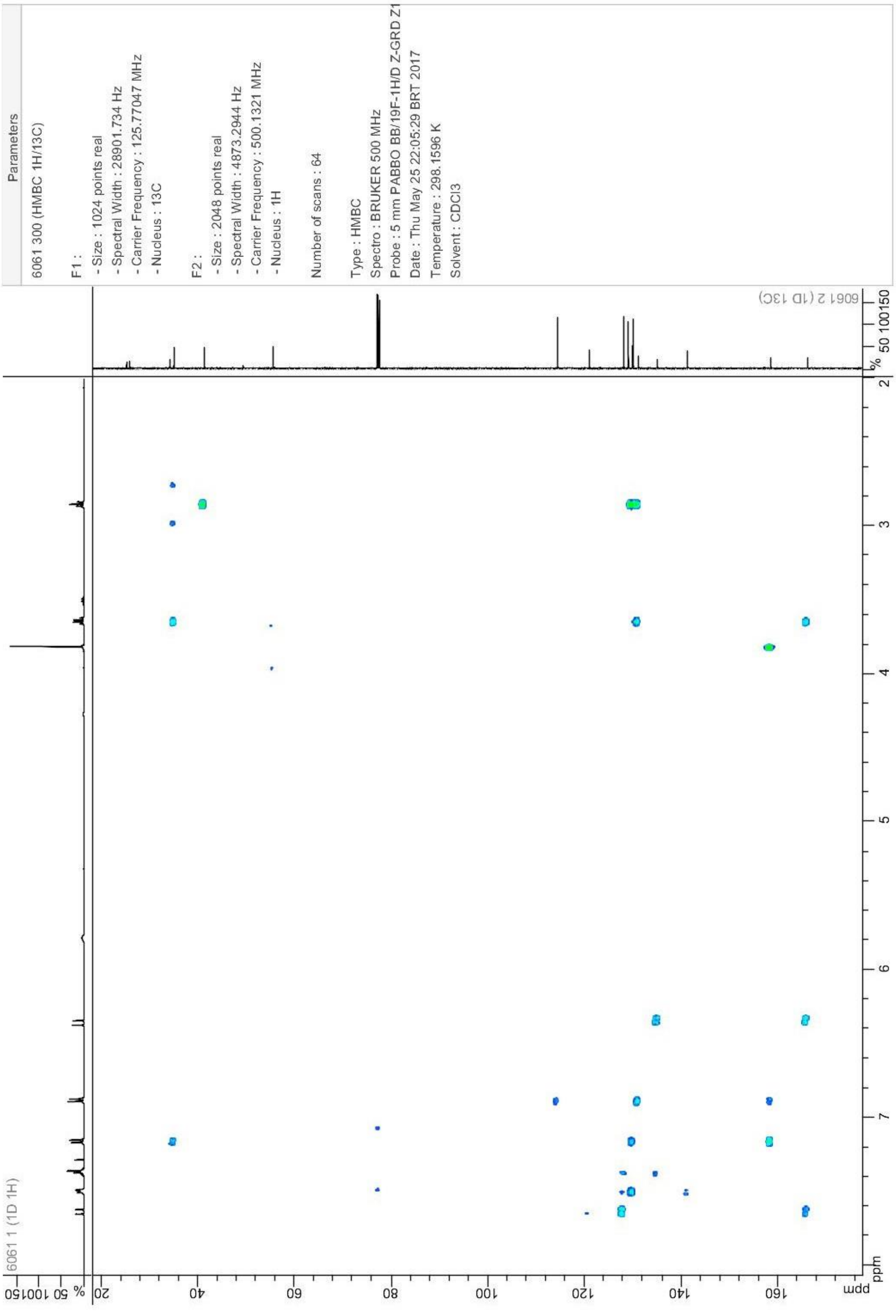


**Figure S3.** HMBC spectrum of compound **1** ($CDCl_3$, 500 MHz).

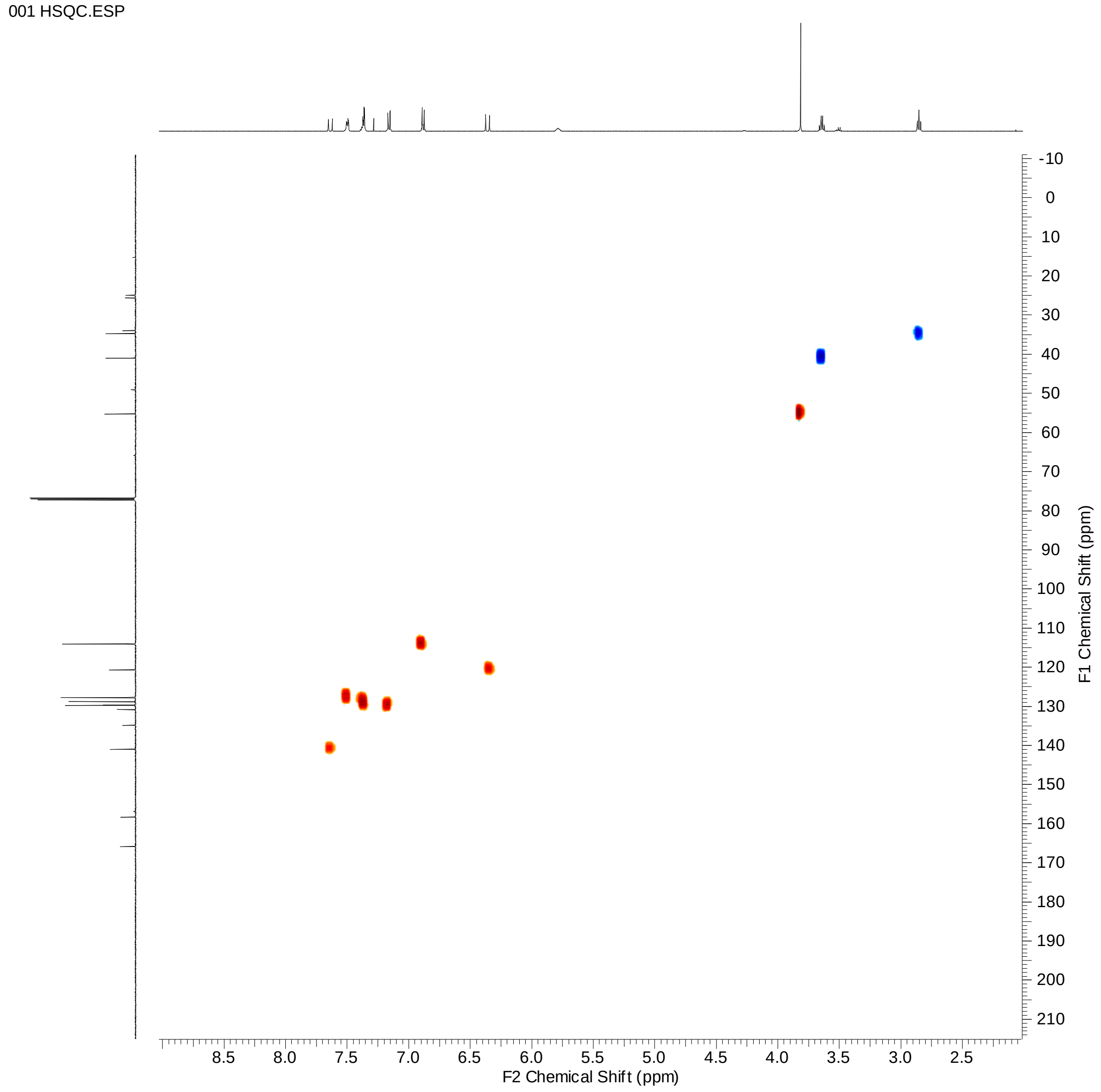


**Figure S4.** HSQC spectrum of compound **1** ($CDCl_3$, 500 MHz).

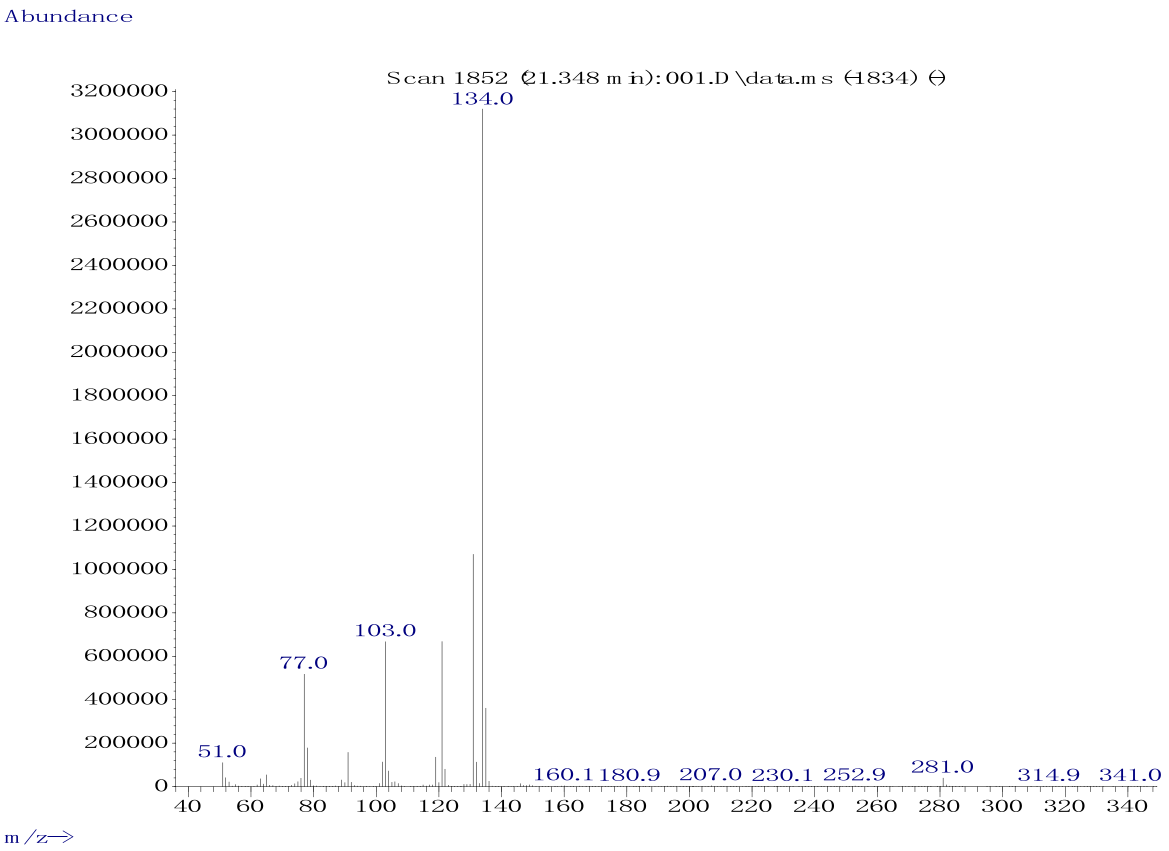


**Figure S5.** Mass spectrum of compound **1** ($CH_2Cl_2$).

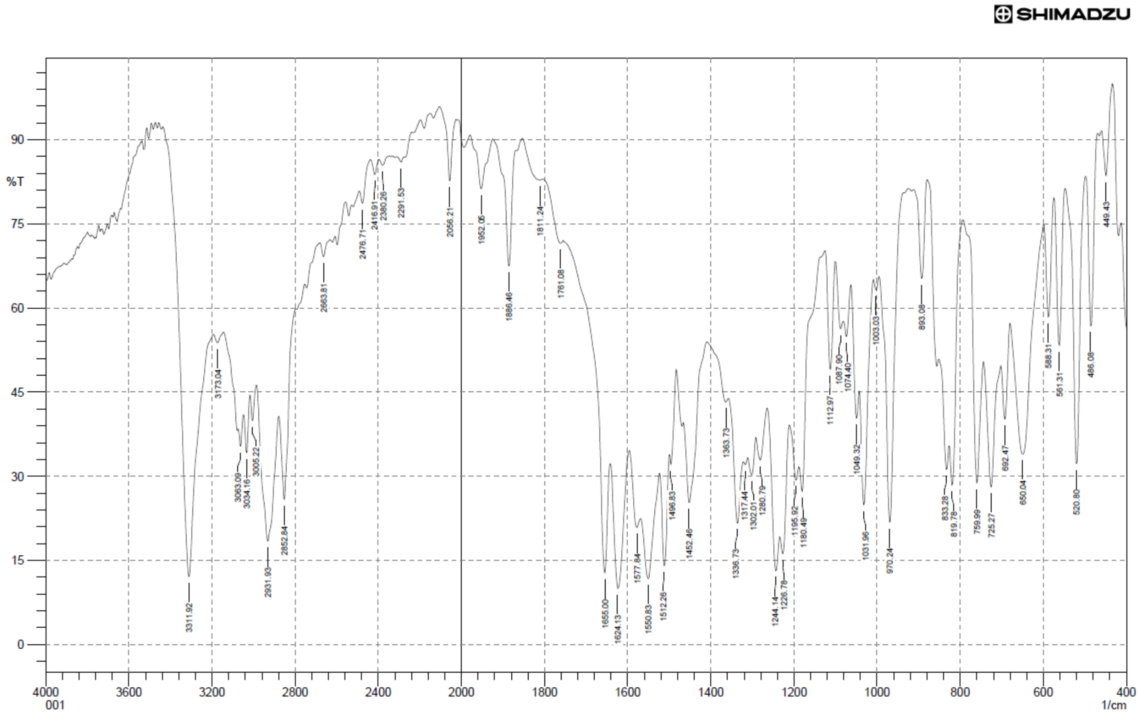


**Figure S6.** IR spectrum (KBr) of compound **1**.

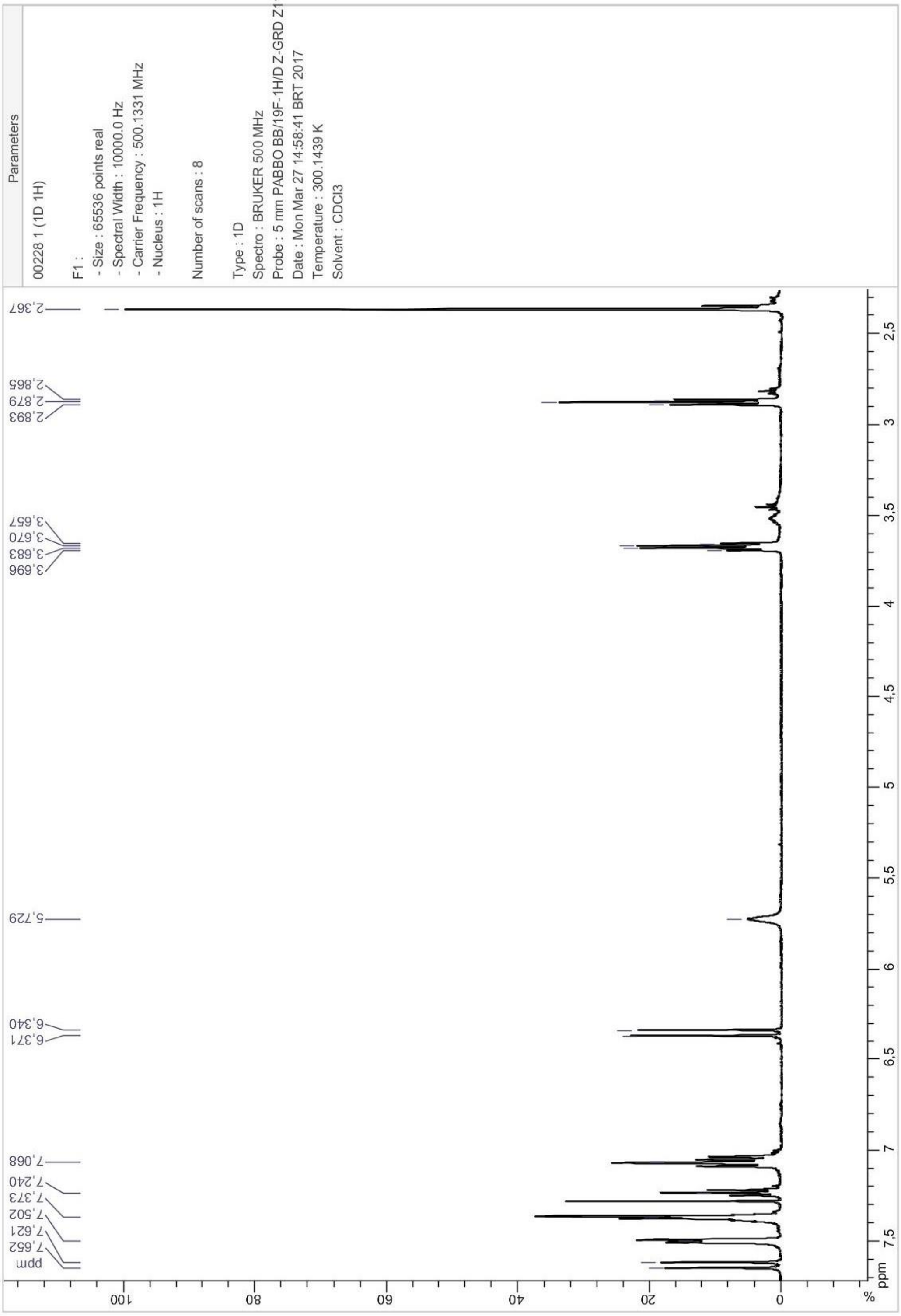


**Figure S7.** $^{1}H$ NMR spectrum of compound **2** ($CDCl_3$, 500 MHz).

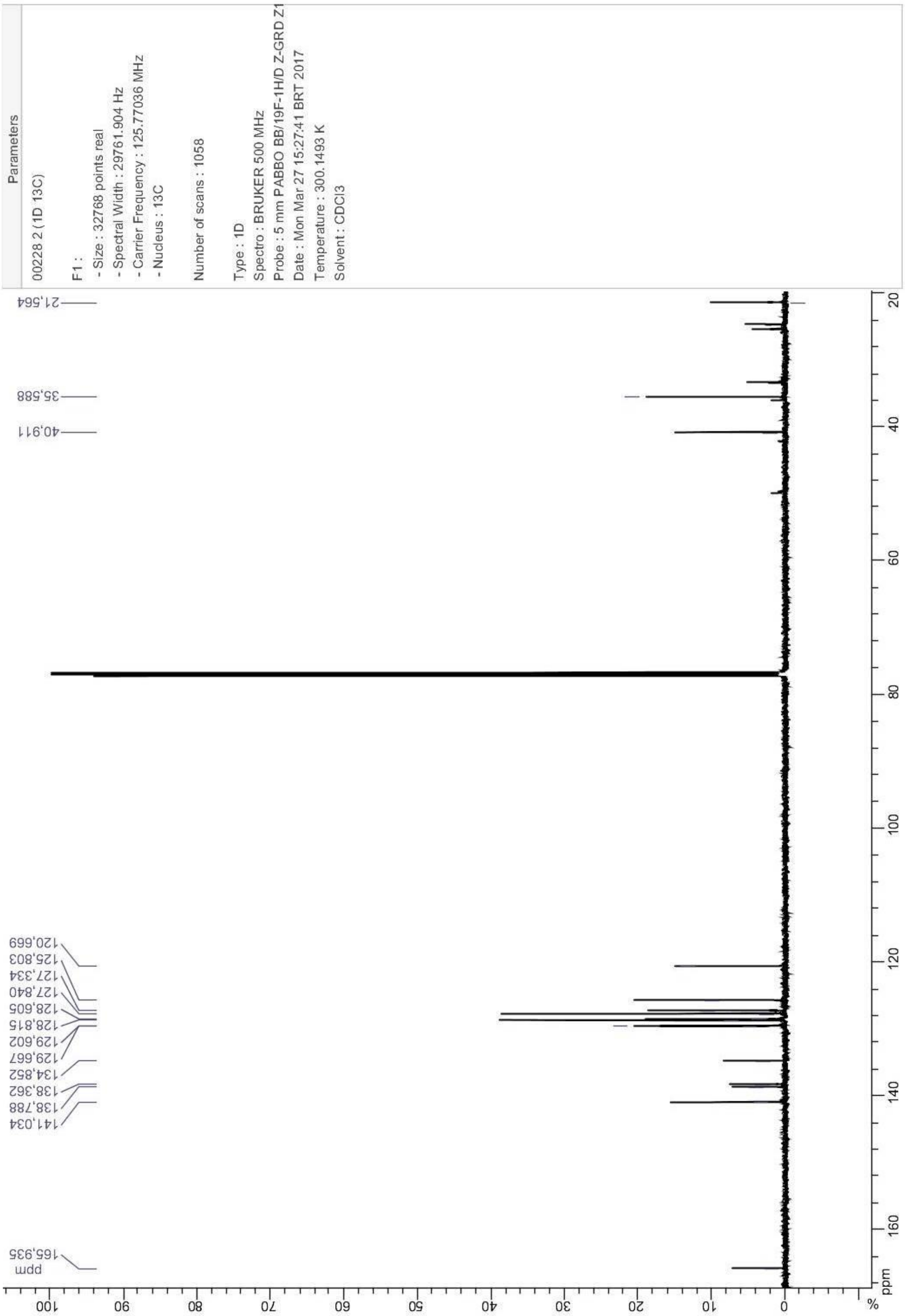


**Figure S8.** $^{13}C$ NMR spectrum of compound **2** ($CDCl_3$, 125 MHz).

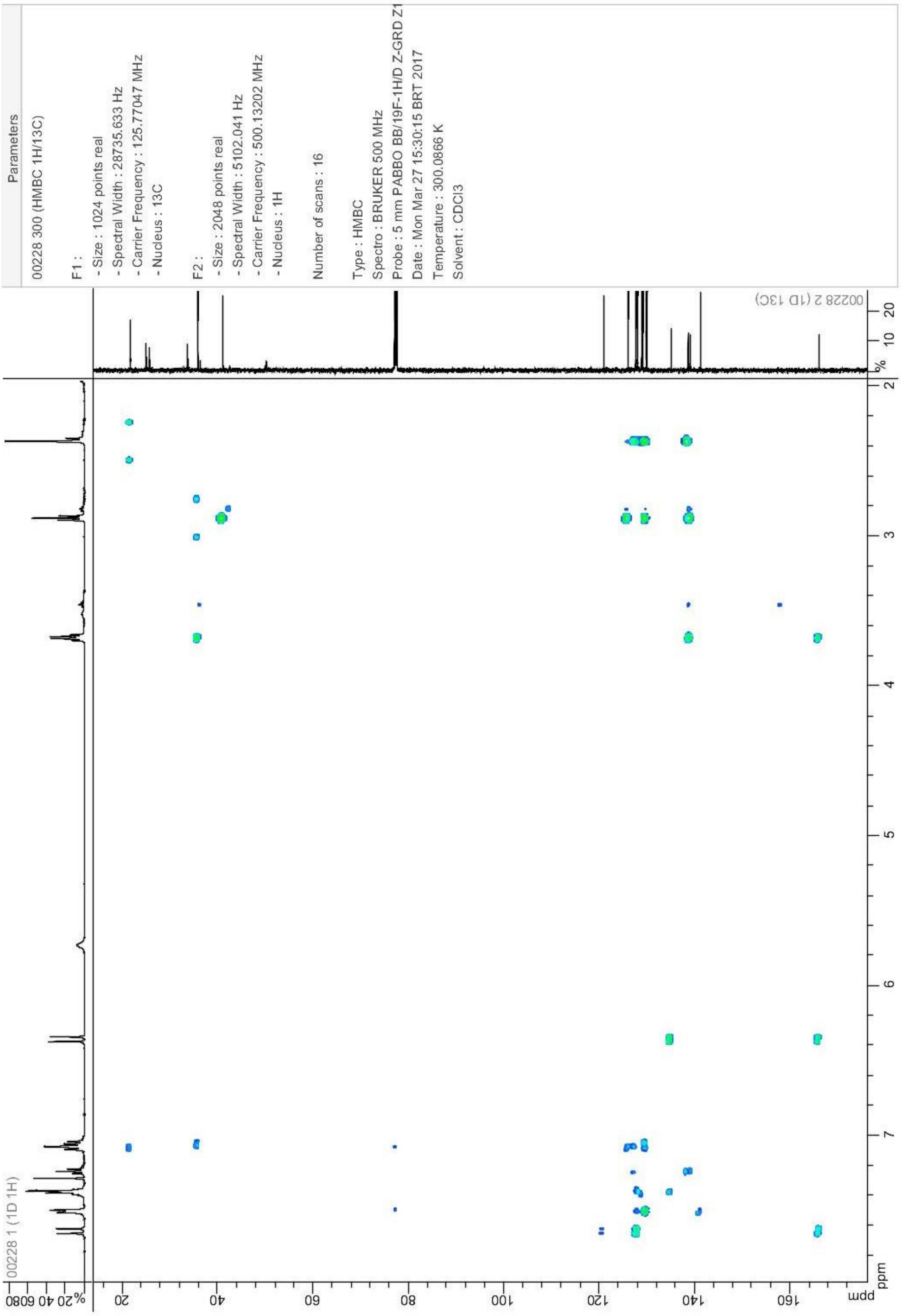


**Figure S9.** HMBC spectrum of compound **2** ($CDCl_3$, 500 MHz).

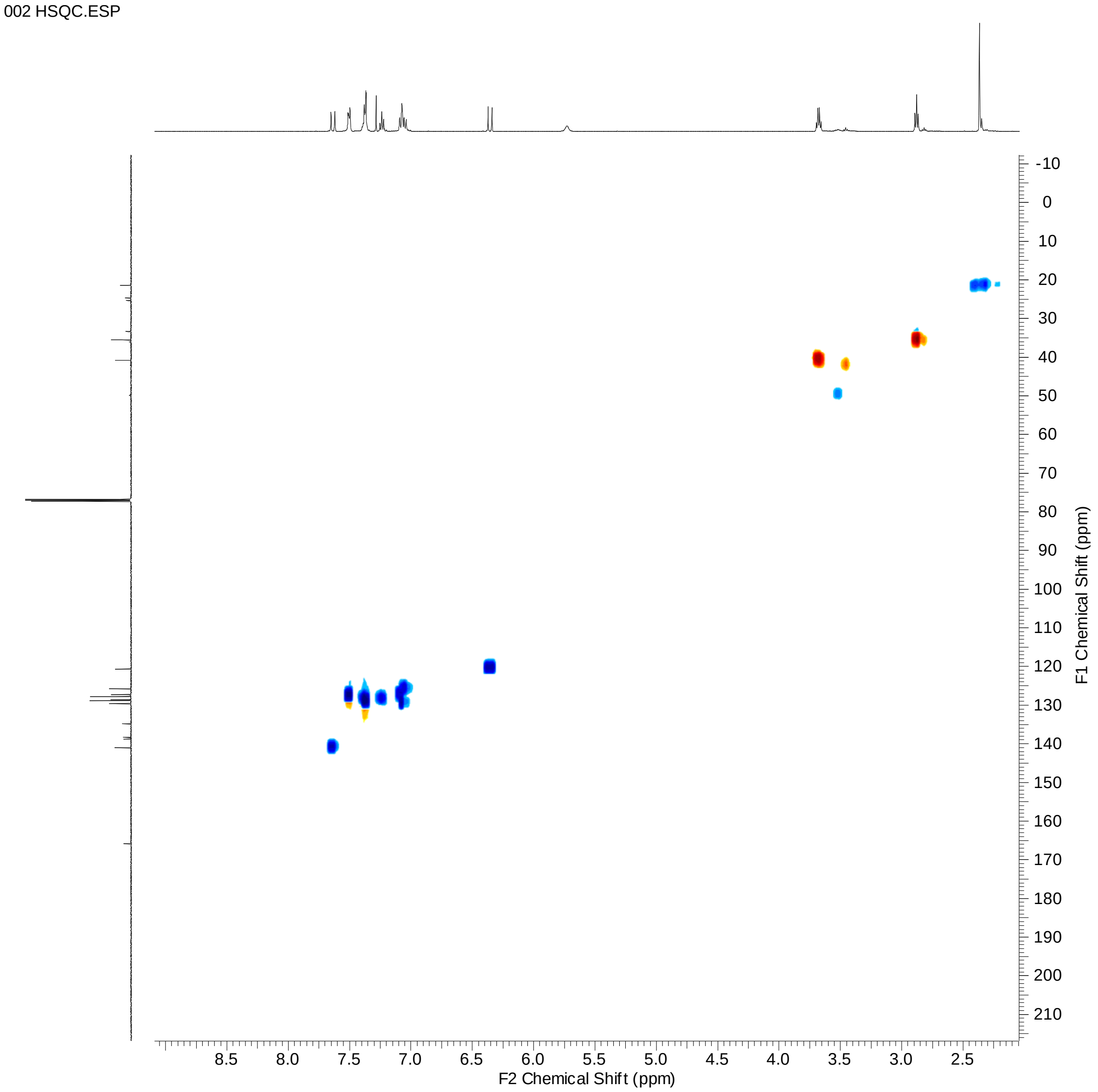


**Figure S10.** HSQC spectrum of compound **2** ($CDCl_3$, 500 MHz).

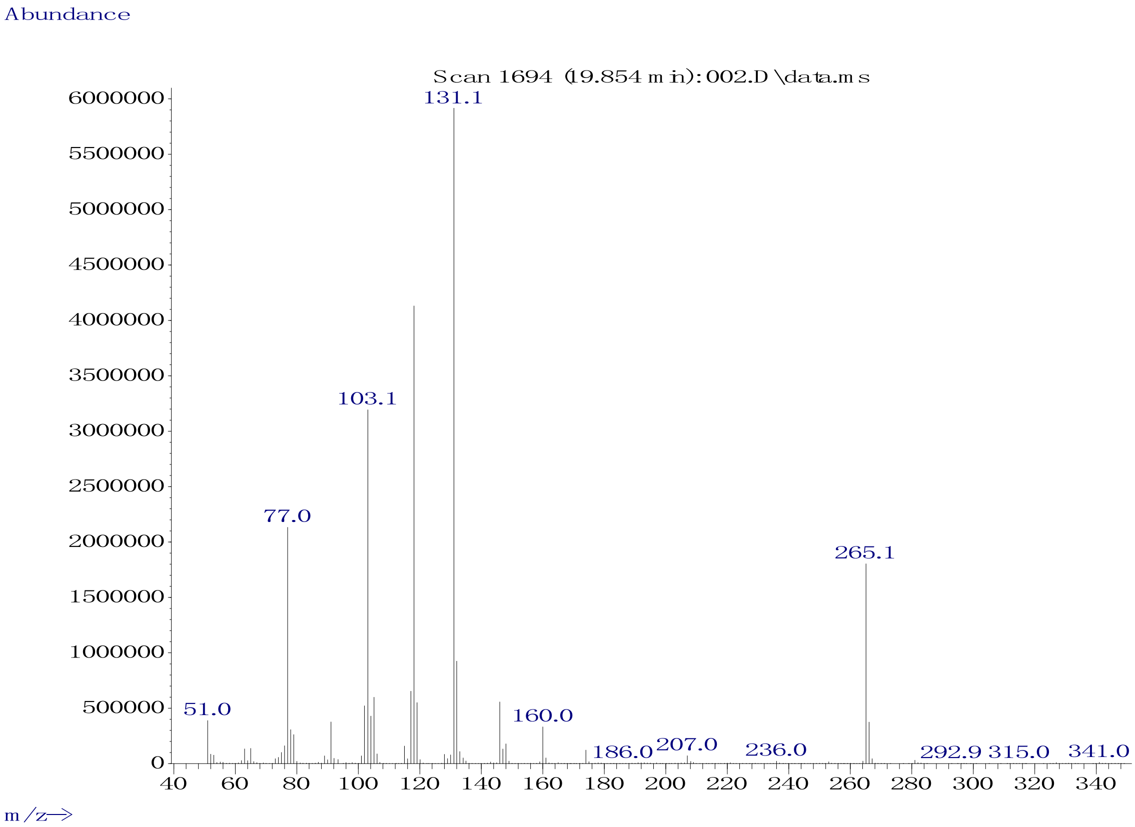


**Figure S11.** Mass spectrum of compound **2** ($CH_2Cl_2$).

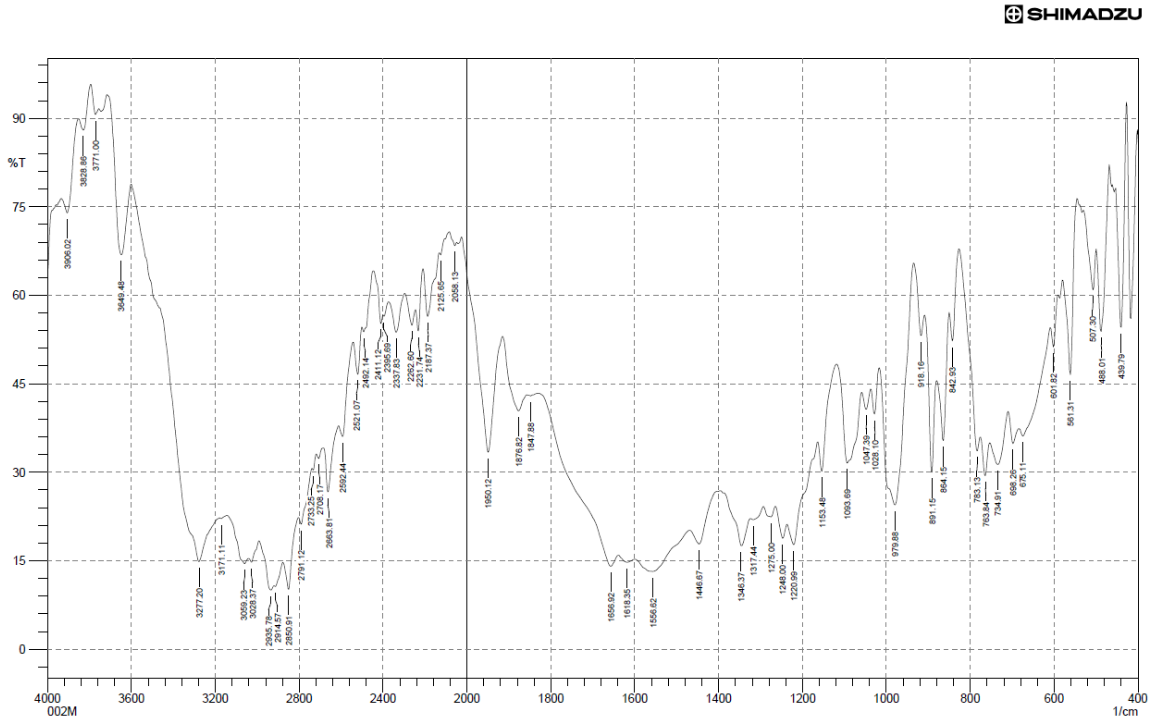


**Figure S12.** IR spectrum (KBr) of compound **2**.

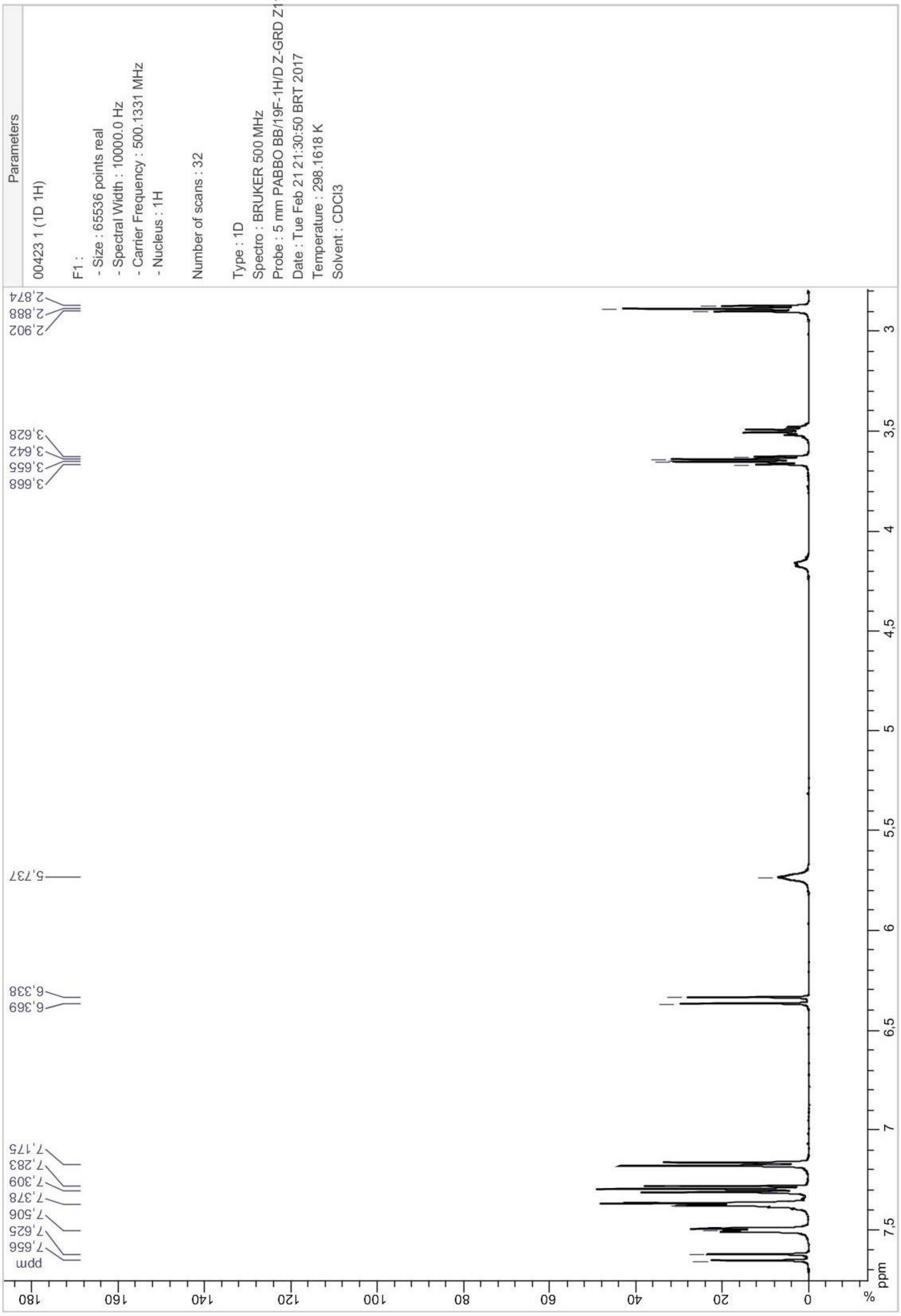


**Figure 13.** $^1$H NMR spectrum of compound **3** ($CDCl_3$, 500 MHz).

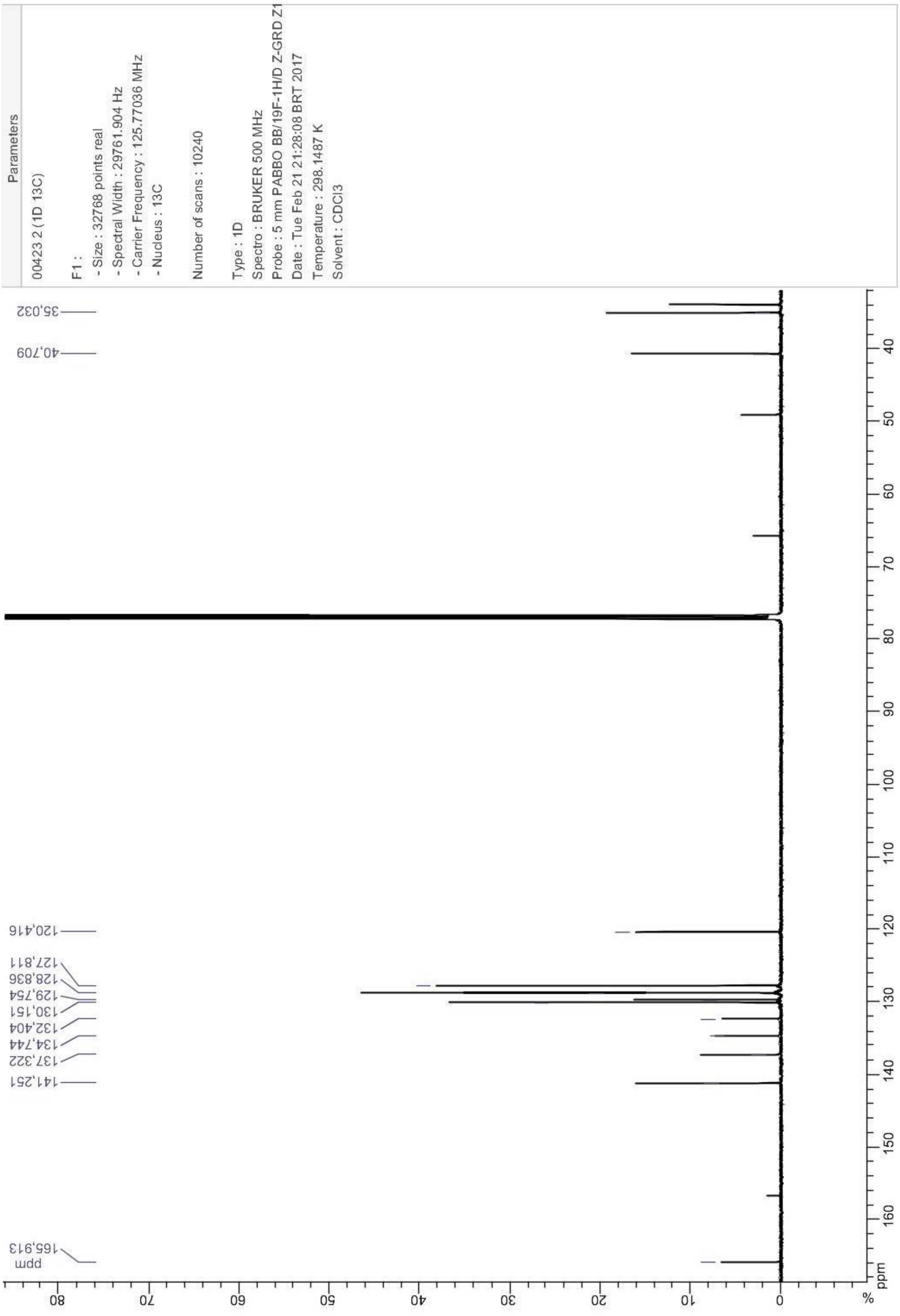


**Figure S14.** $^{13}C$ NMR spectrum of compound **3** ($CDCl_3$, 125 MHz).

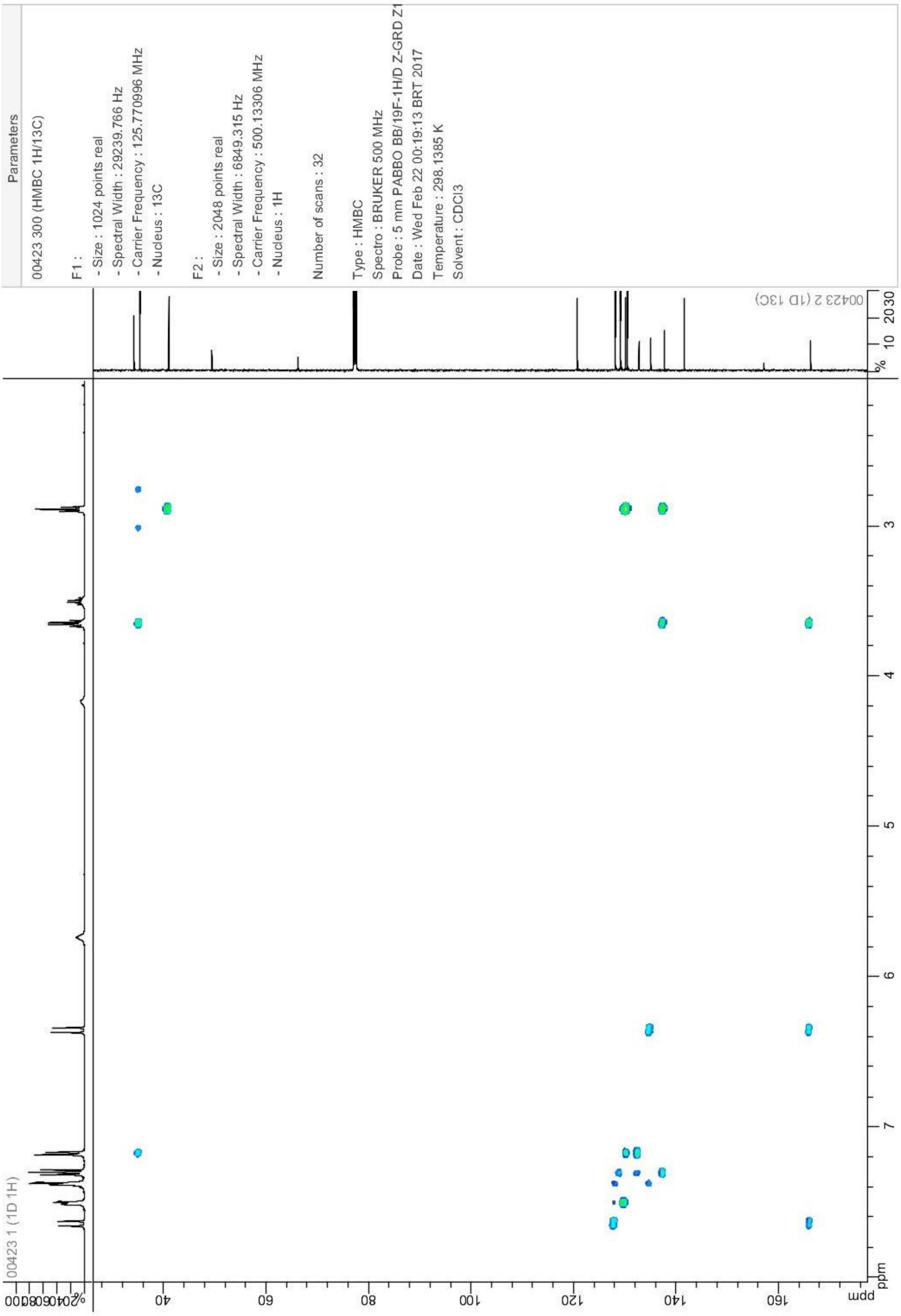


**Figure S15.** HMBC spectrum of compound **3** ($CDCl_3$, 500 MHz).

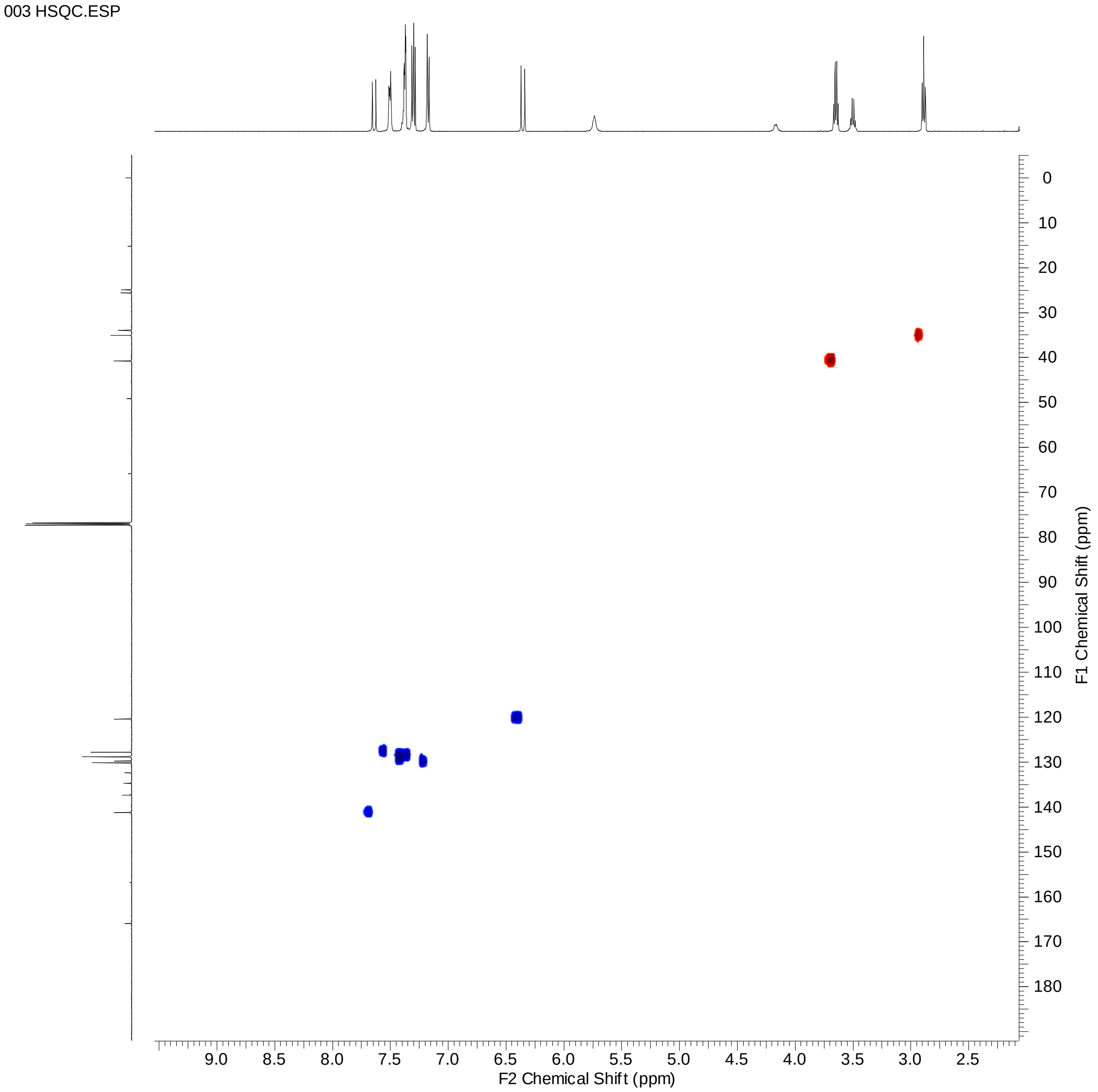


**Figure S16** HSQC spectrum of compound **3** ($CDCl_3$, 500 MHz).

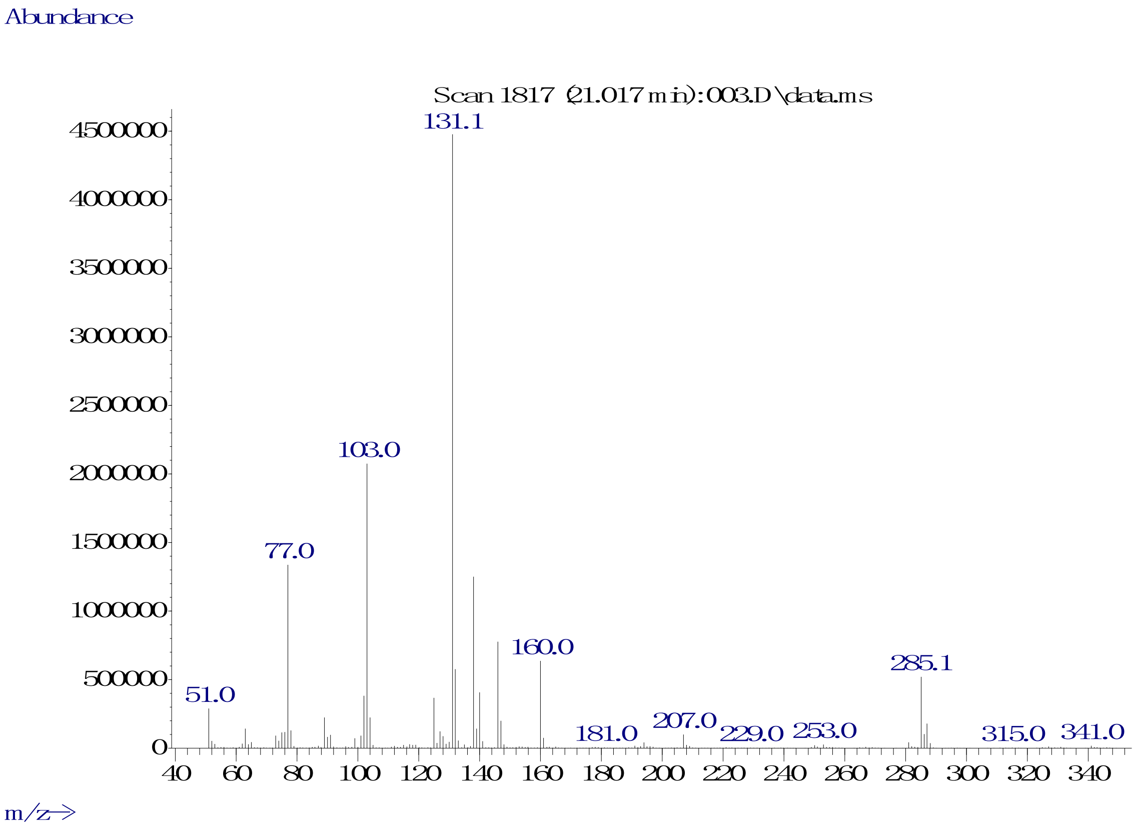


**Figure S17.** Mass spectrum of compound **3** ($CH_2Cl_2$).

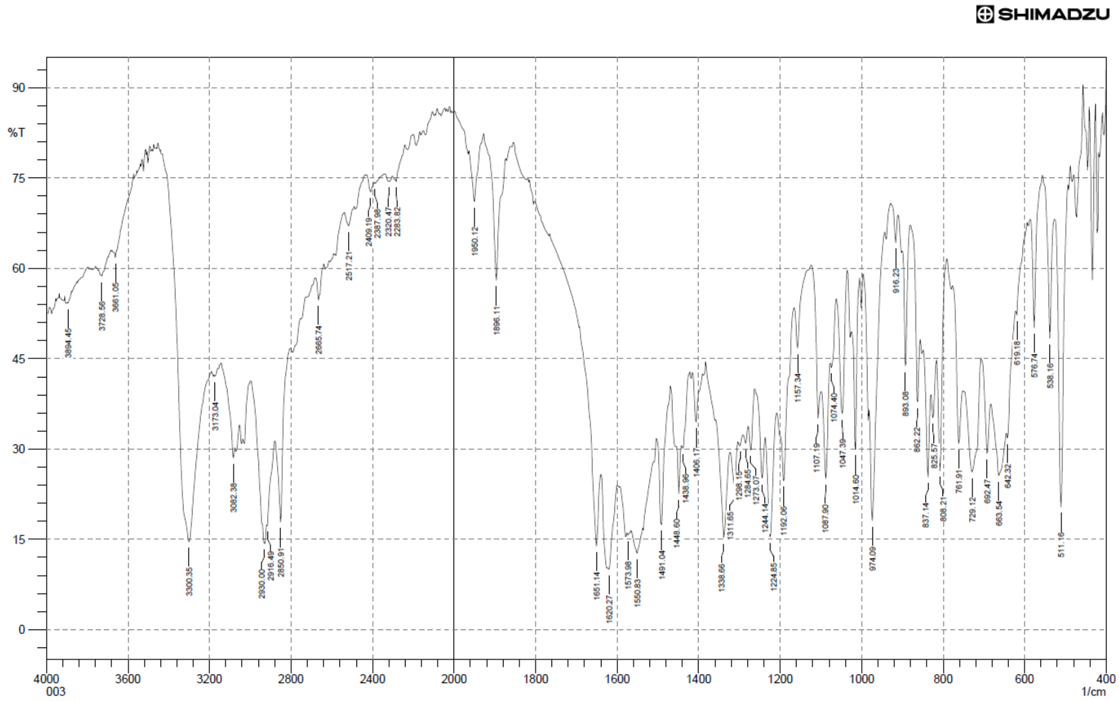


**Figure S18.** IR spectrum (KBr) of compound **3**.

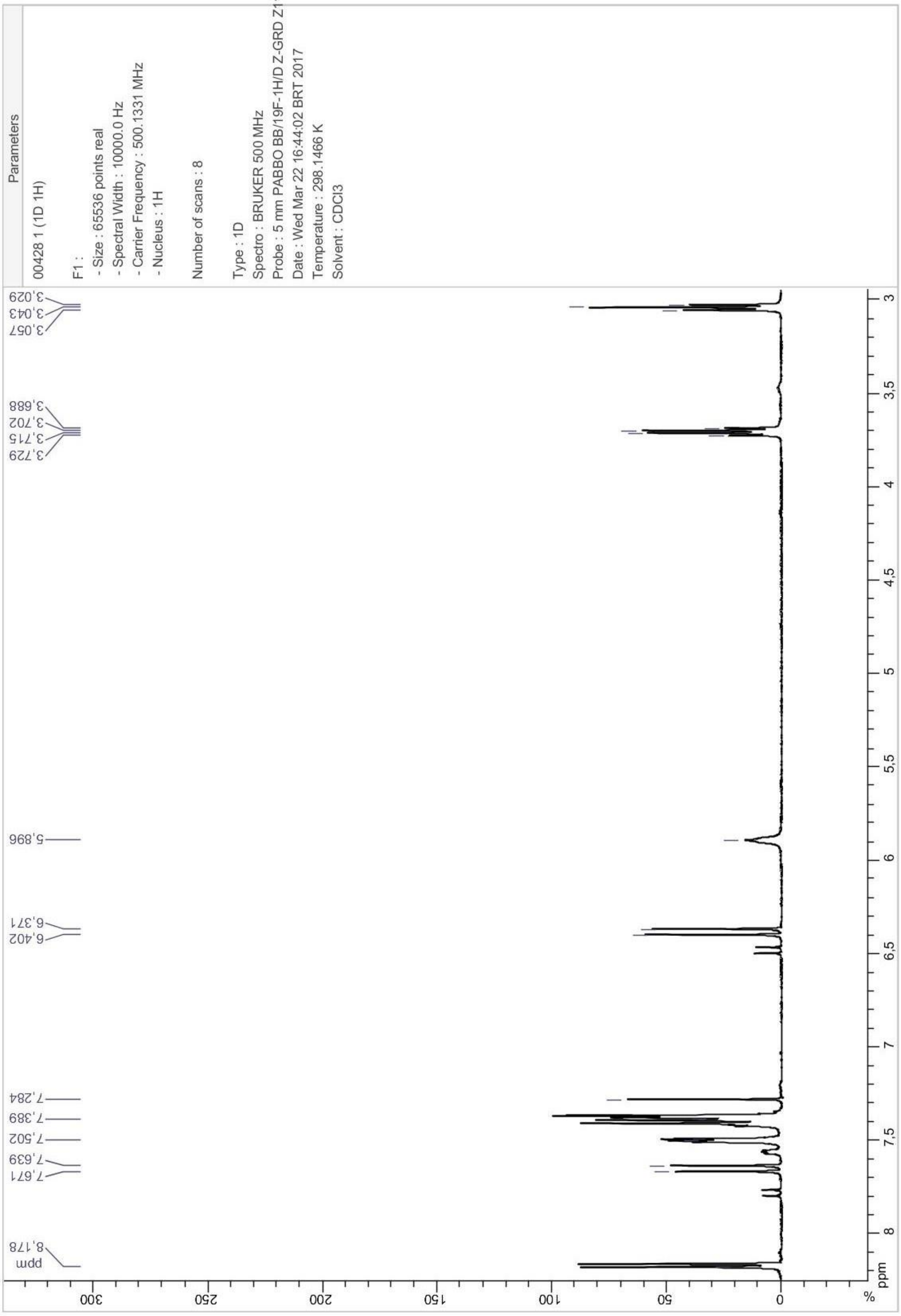


**Figure S19.** $^{1}$H NMR spectrum of compound **4** ($CDCl_3$, 500 MHz).

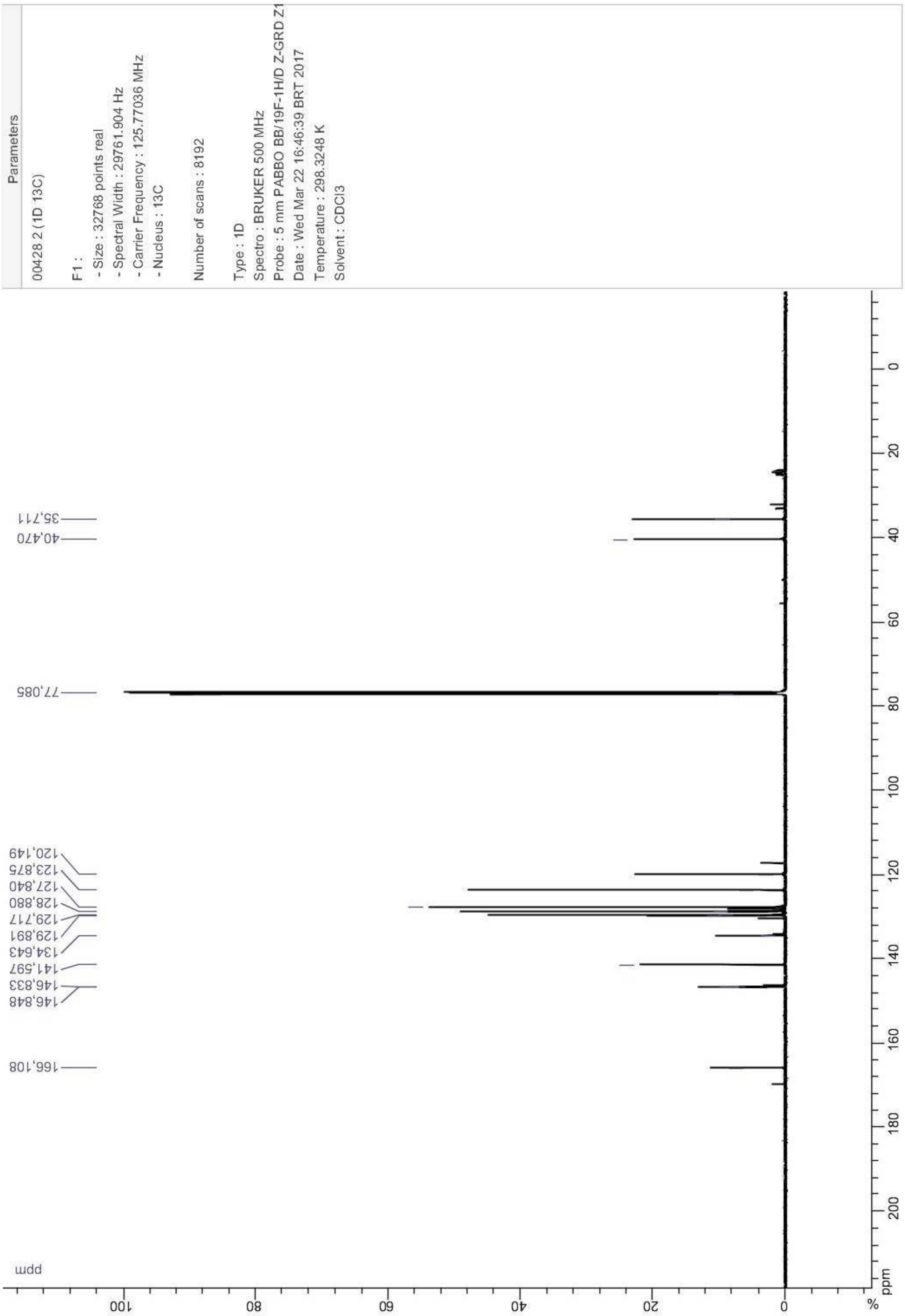


**Figure S20.** $^{13}C$ NMR spectrum of compound **4** ($CDCl_3$, 125 MHz).

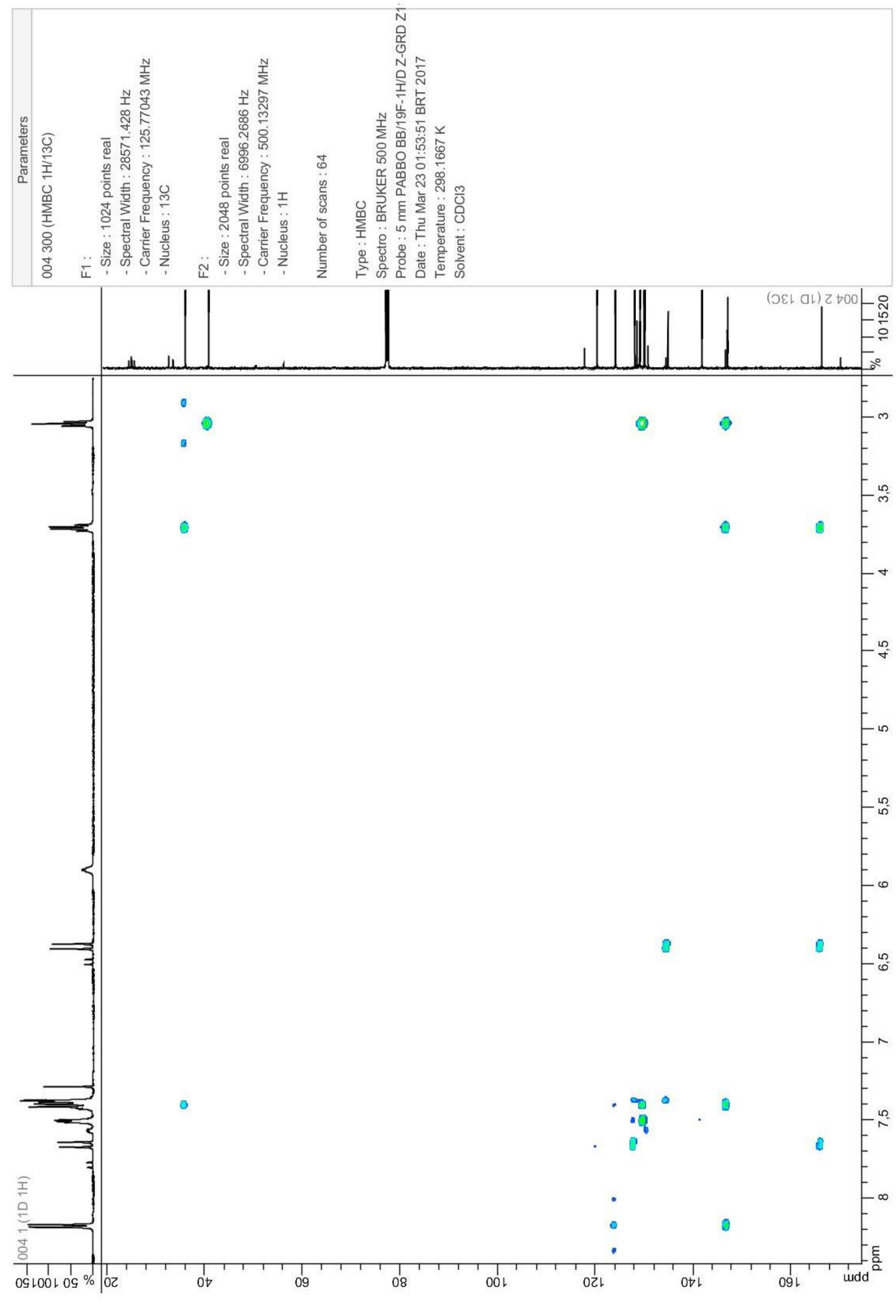


**Figure S21.** HMBC spectrum of compound **4** ($CDCl_3$, 500 MHz).

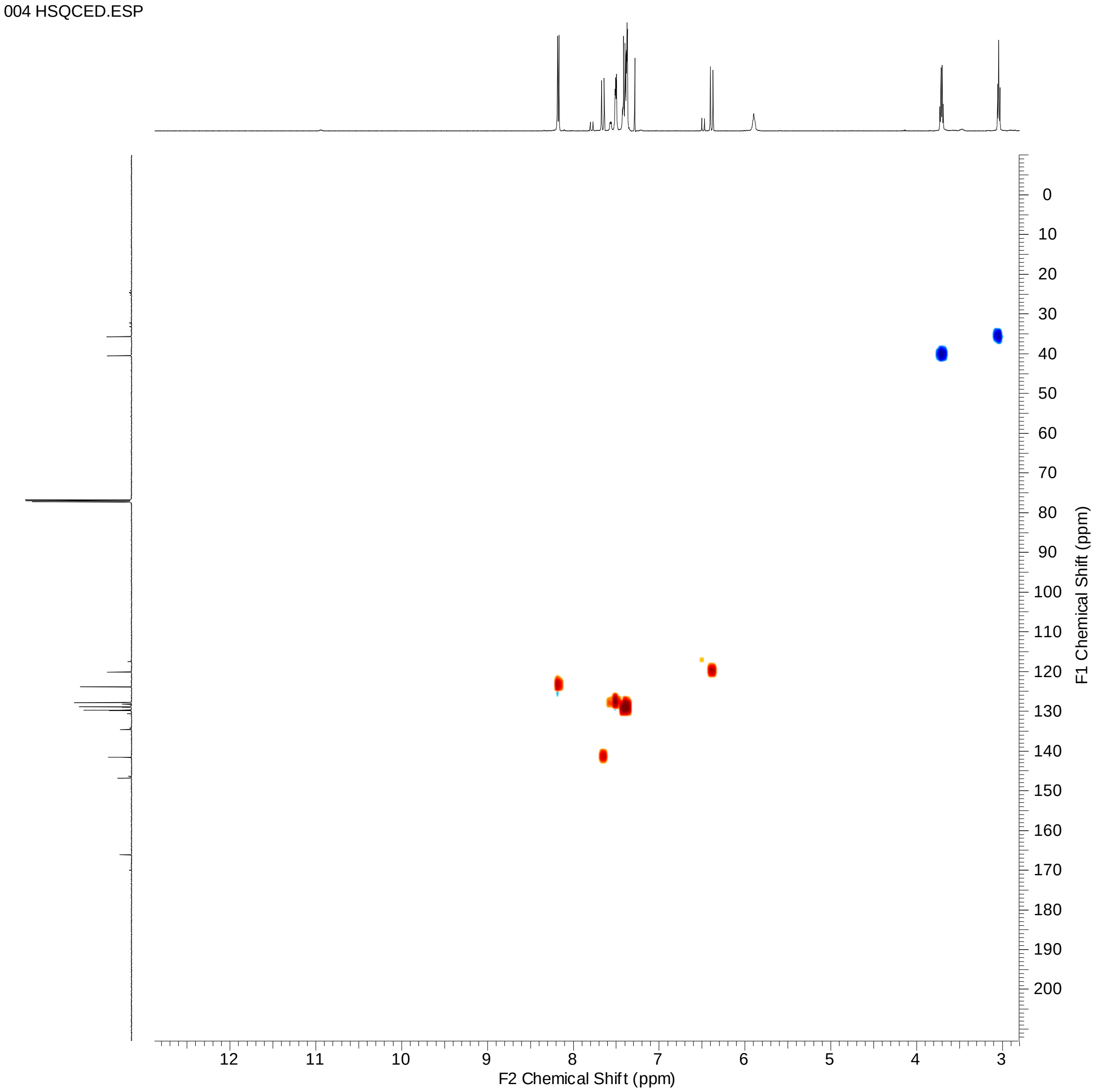


**Figure S22.** HSQC spectrum of compound **4** ($CDCl_3$, 500 MHz).

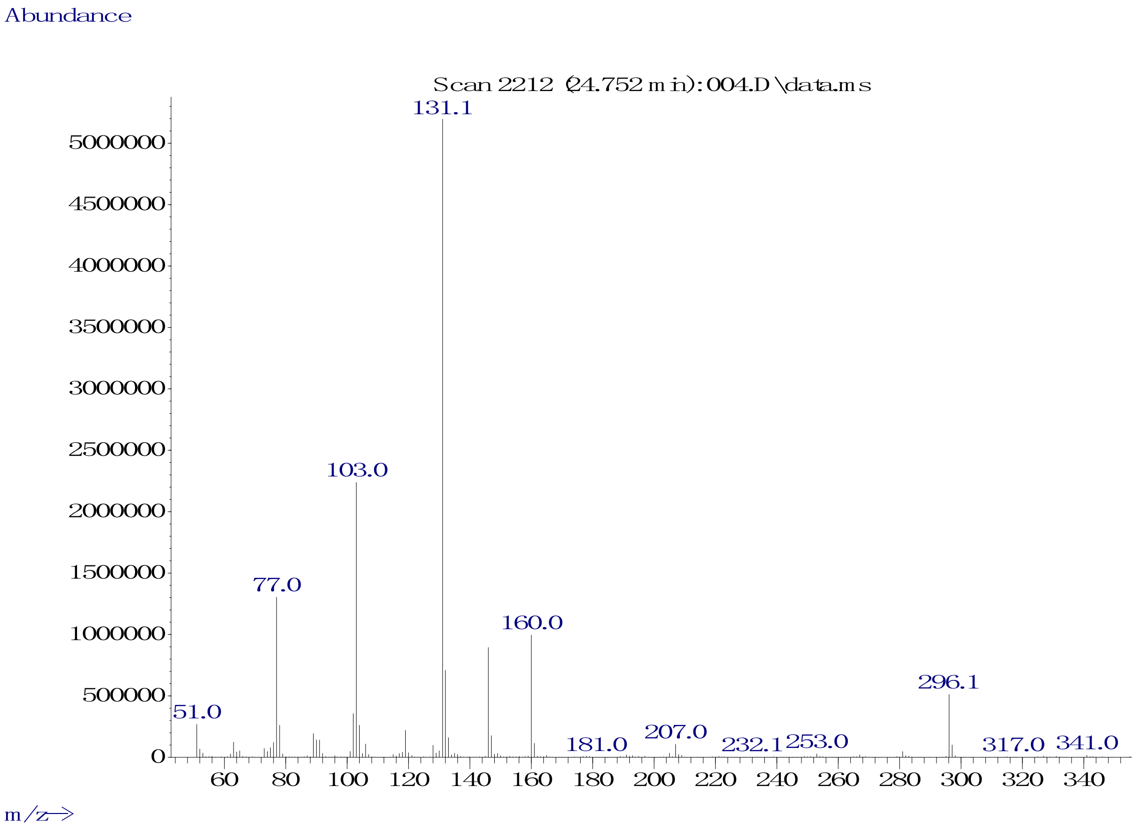


**Figure S23.** Mass spectrum of compound **4** ($CH_2Cl_2$).

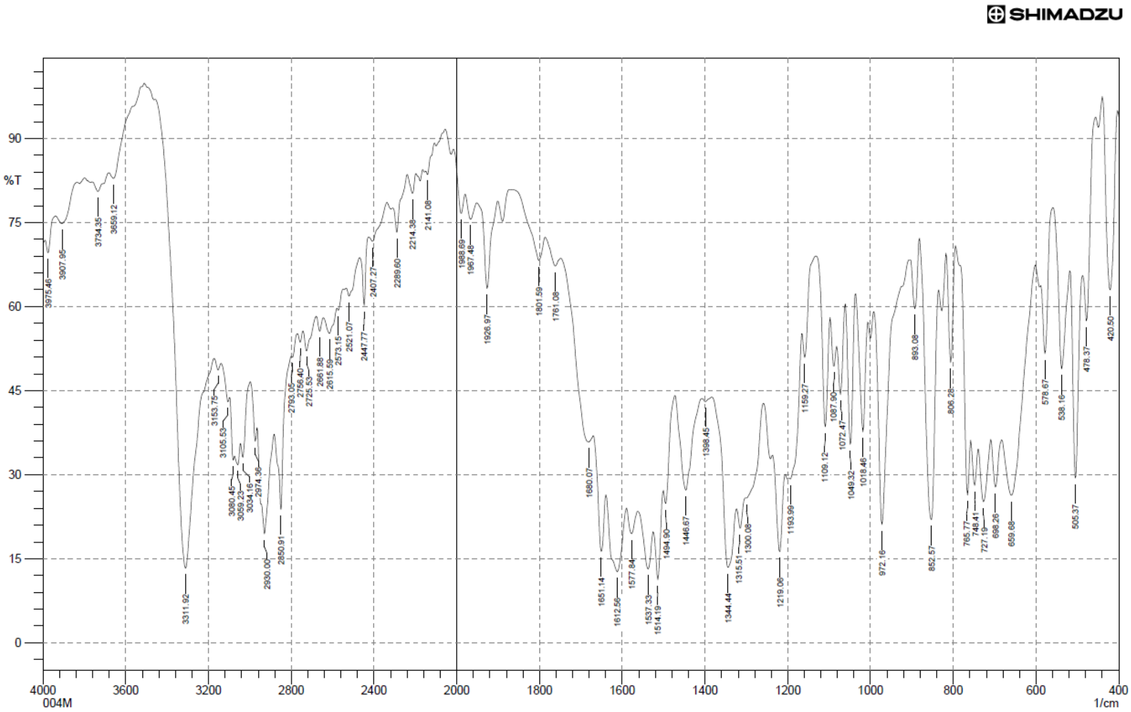


**Figure S24.** IR spectrum (KBr) of compound **4**.

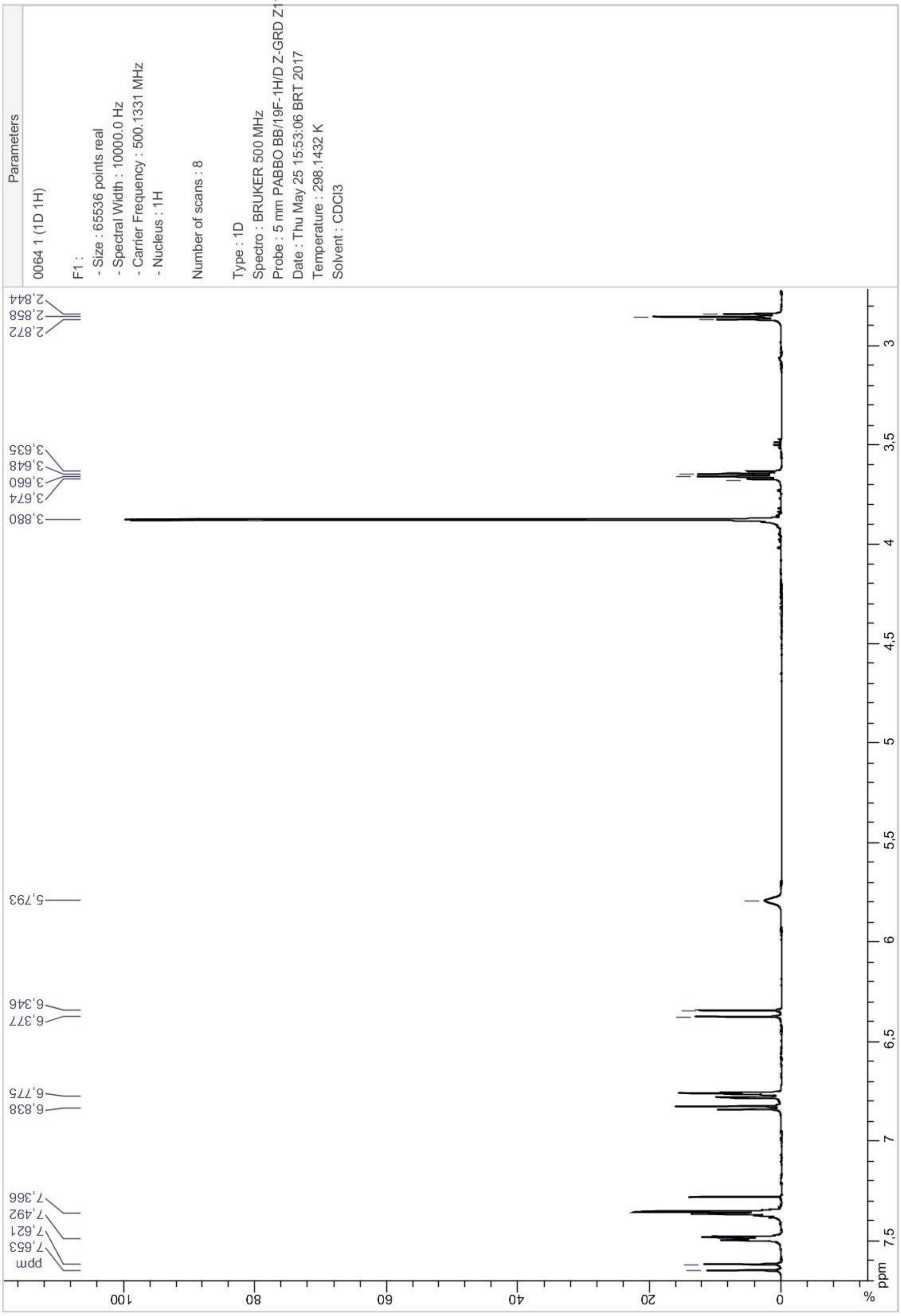


**Figure S25.** ¹H NMR spectrum of compound **5** ($CDCl_3$, 500 MHz).

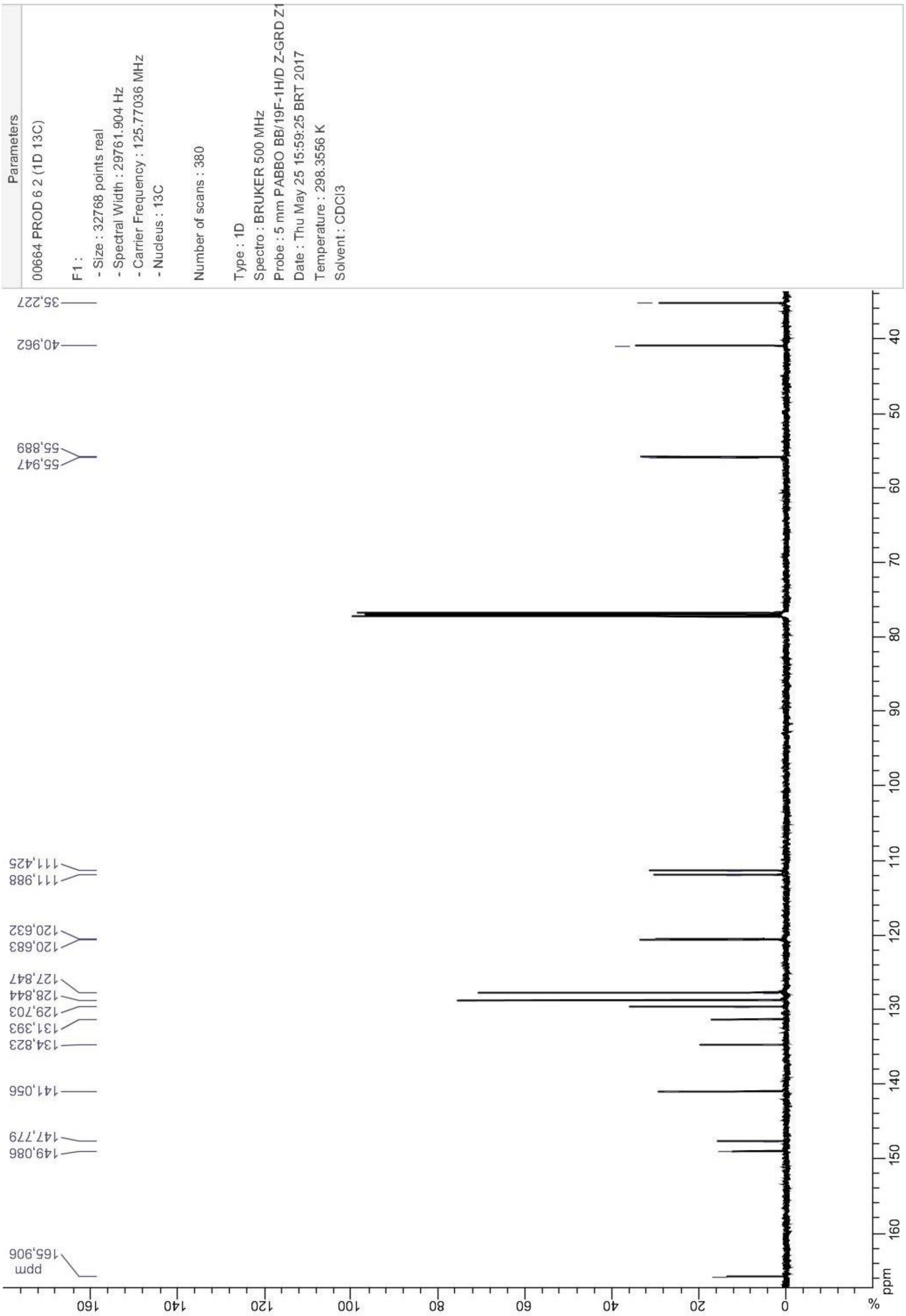


**Figure S26.** $^{13}C$ NMR spectrum of compound **5** ($CDCl_3$, 125 MHz).

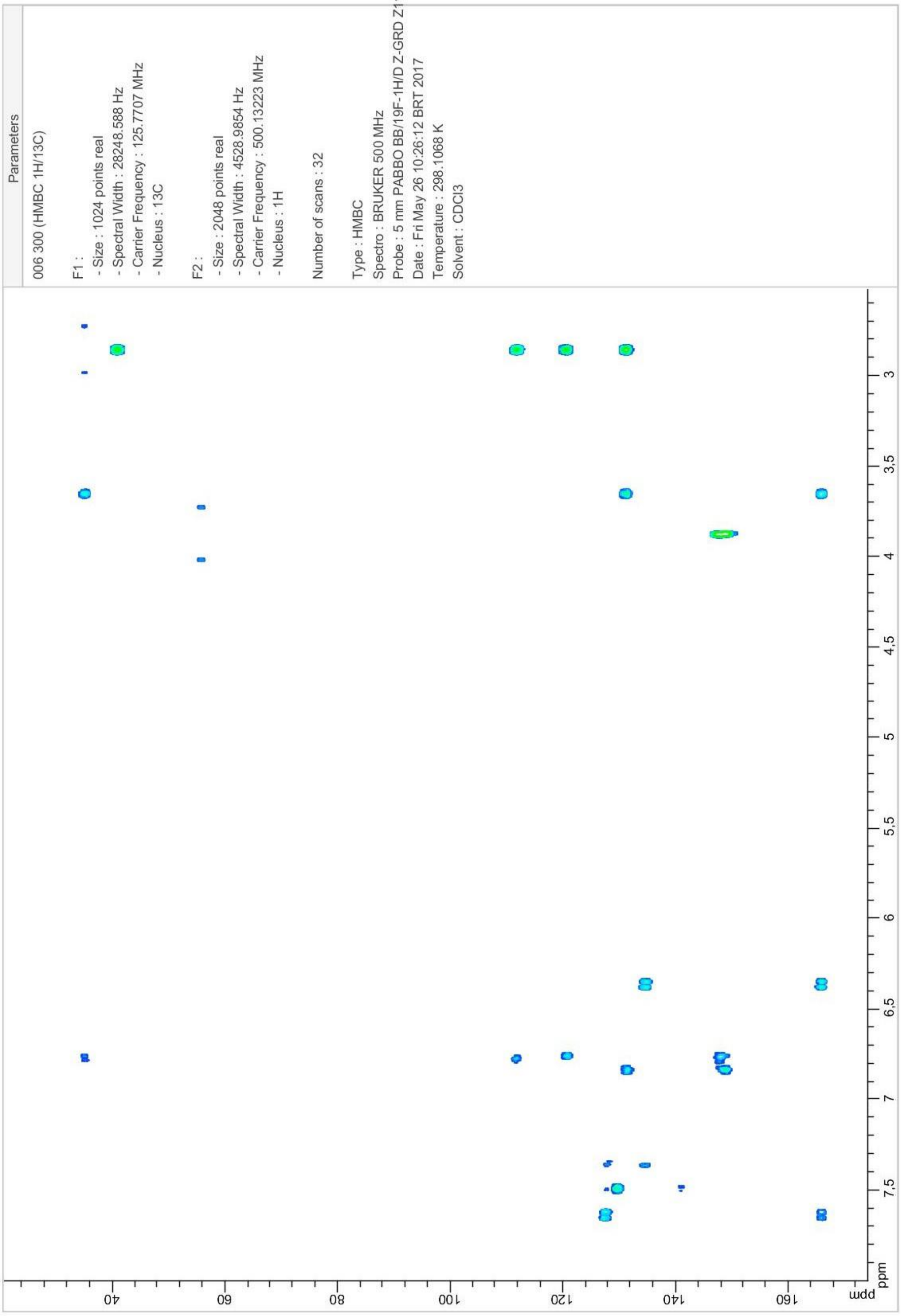


**Figure S27.** HMBC spectrum of compound **5** ($CDCl_3$, 500 MHz).

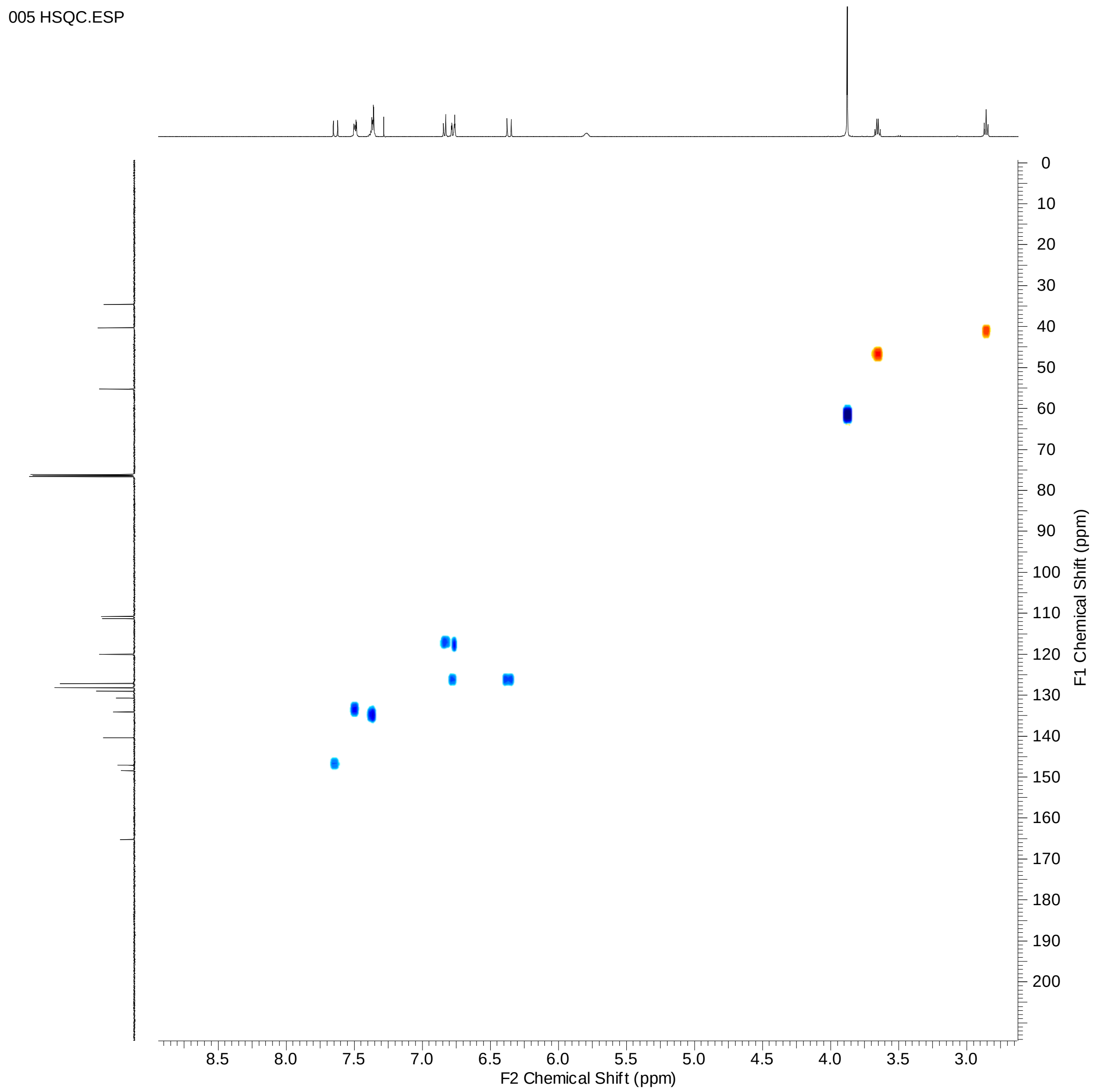


**Figure S28.** HSQC spectrum of compound **5** ($CDCl_3$, 500 MHz).

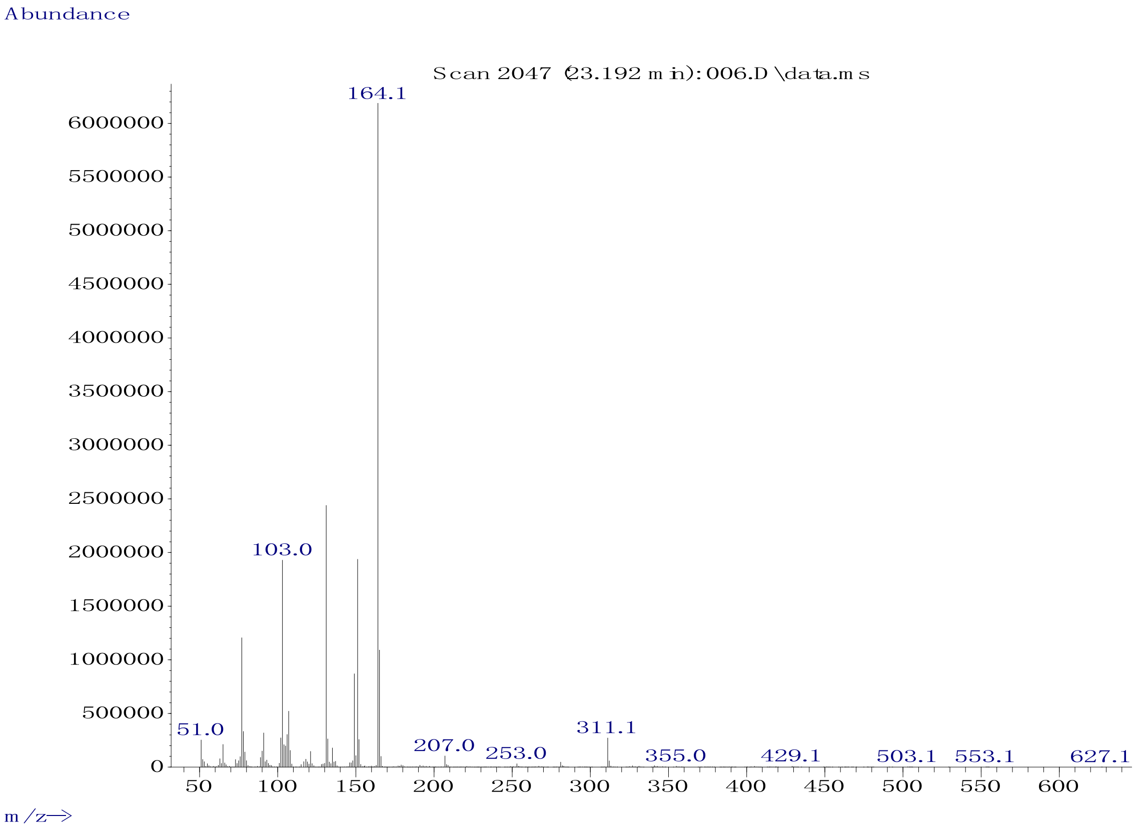


**Figure S29.** Mass spectrum of compound **5** ($CH_2Cl_2$).

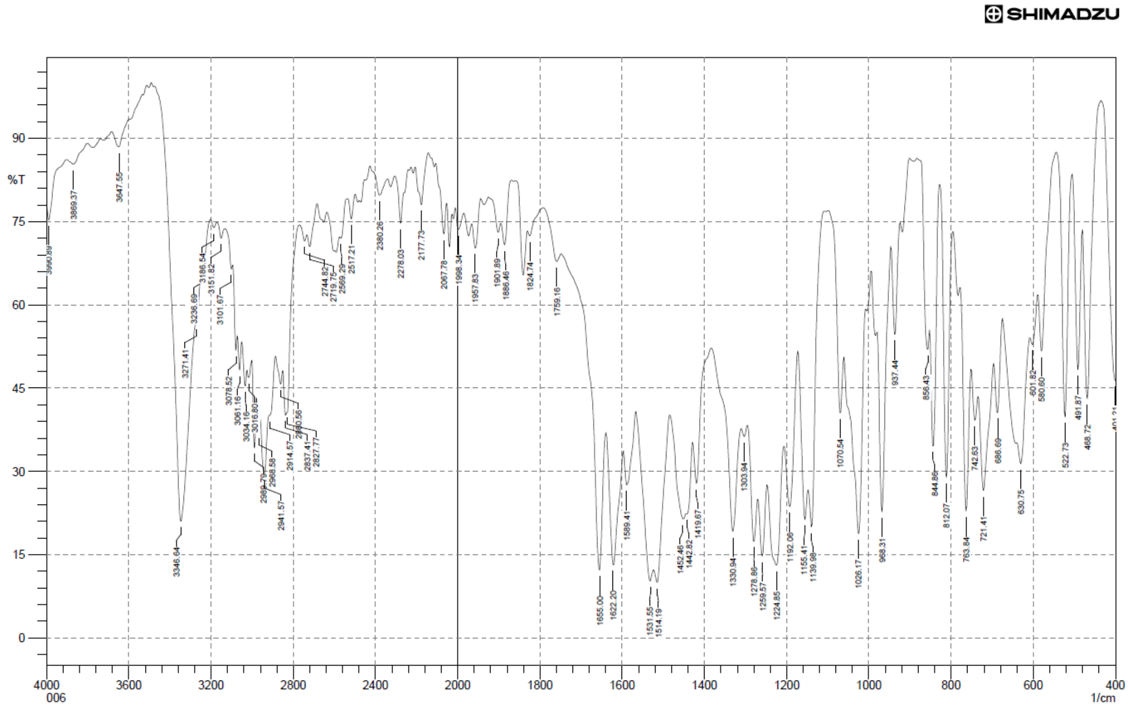


**Figure S30.** IR spectrum (KBr) of compound **5**.

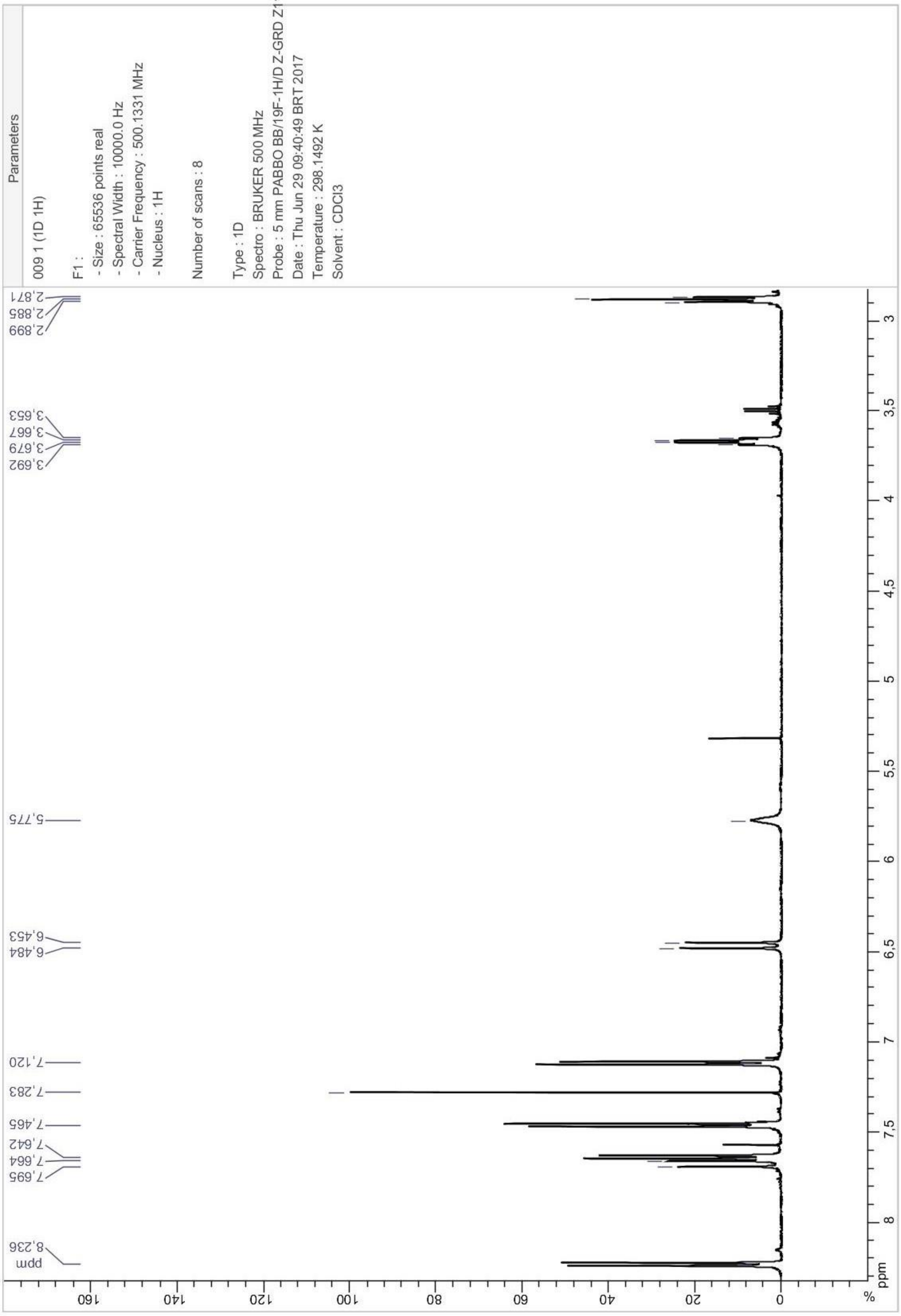


**Figure S31.** $^1$H NMR spectrum of compound **6** ($CDCl_3$, 500 MHz).

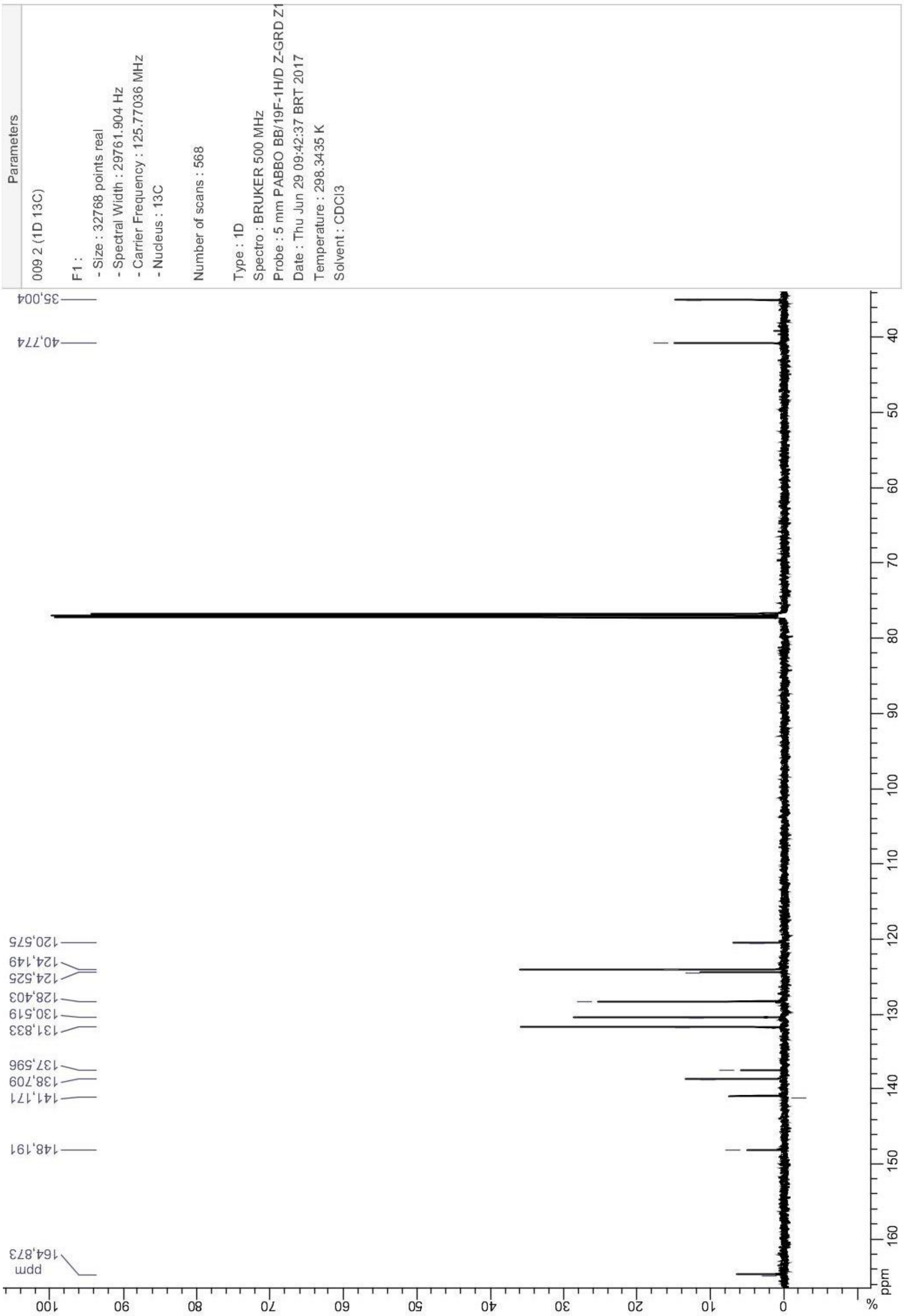


**Figure S32.** $^{13}C$ NMR spectrum of compound **6** ($CDCl_3$, 125 MHz).

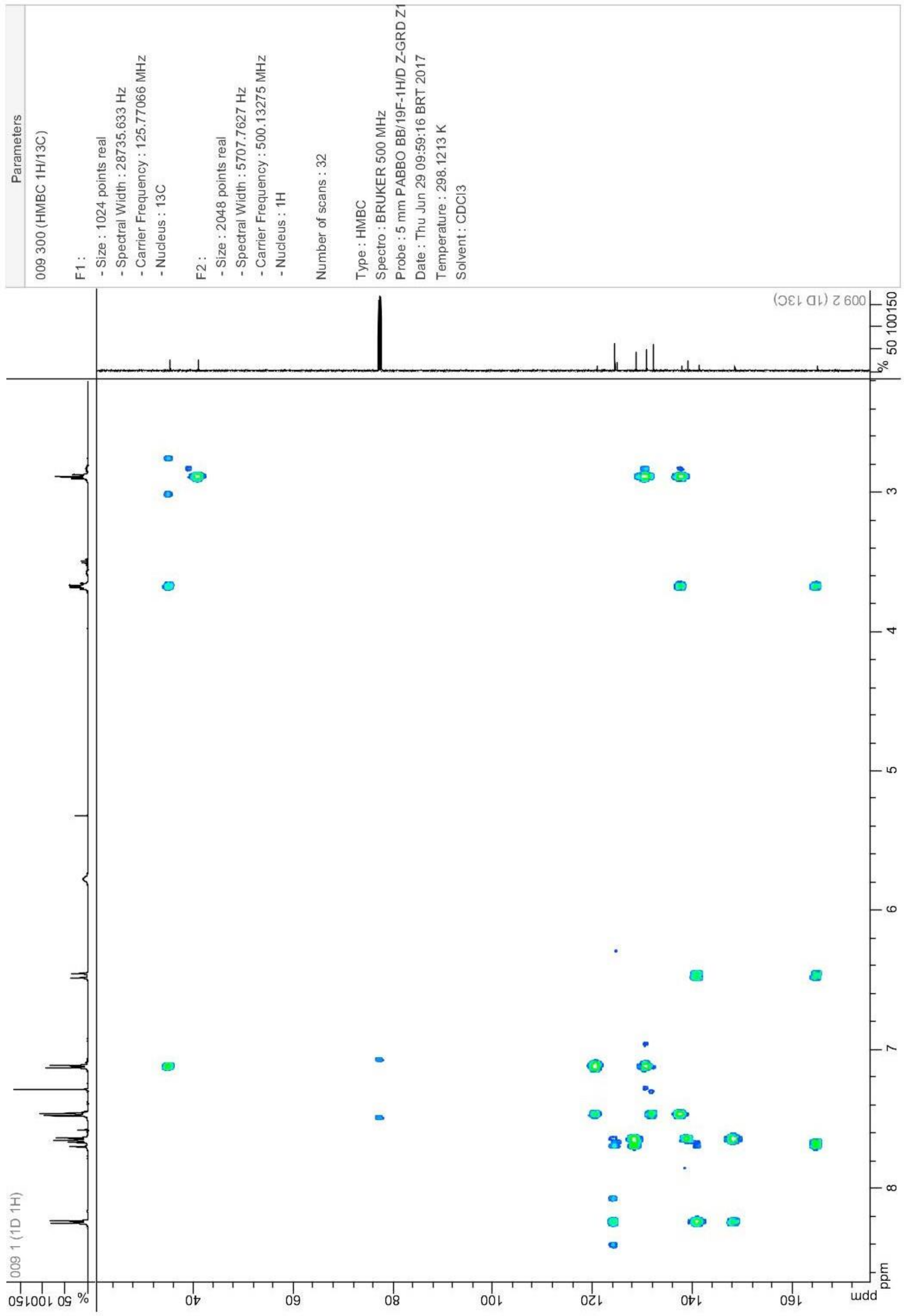


**Figure S33.** HMBC spectrum of compound **6** ($CDCl_3$, 500 MHz).

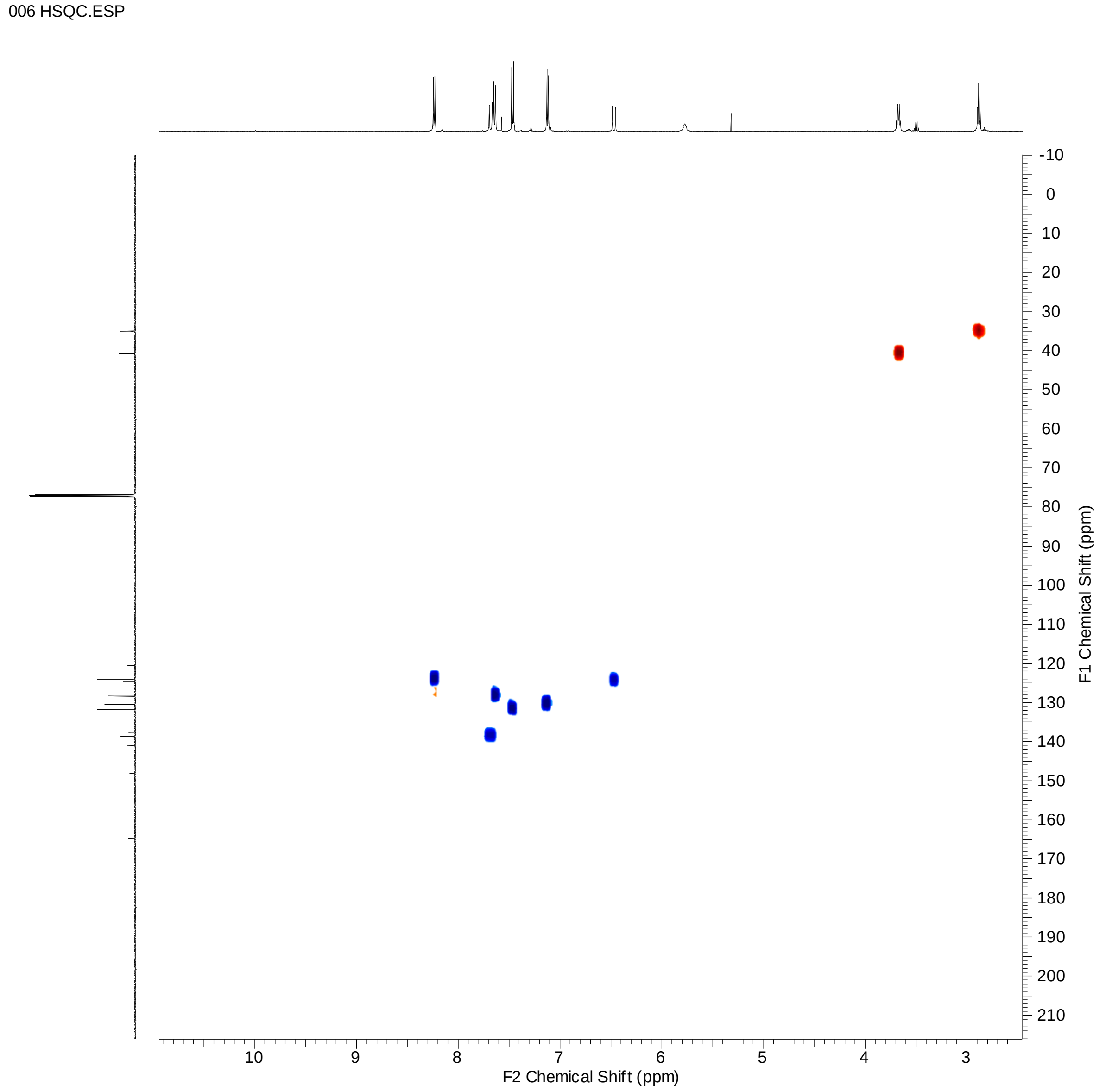


**Figure S34.** HSQC spectrum of compound **6** ($CDCl_3$, 500 MHz).

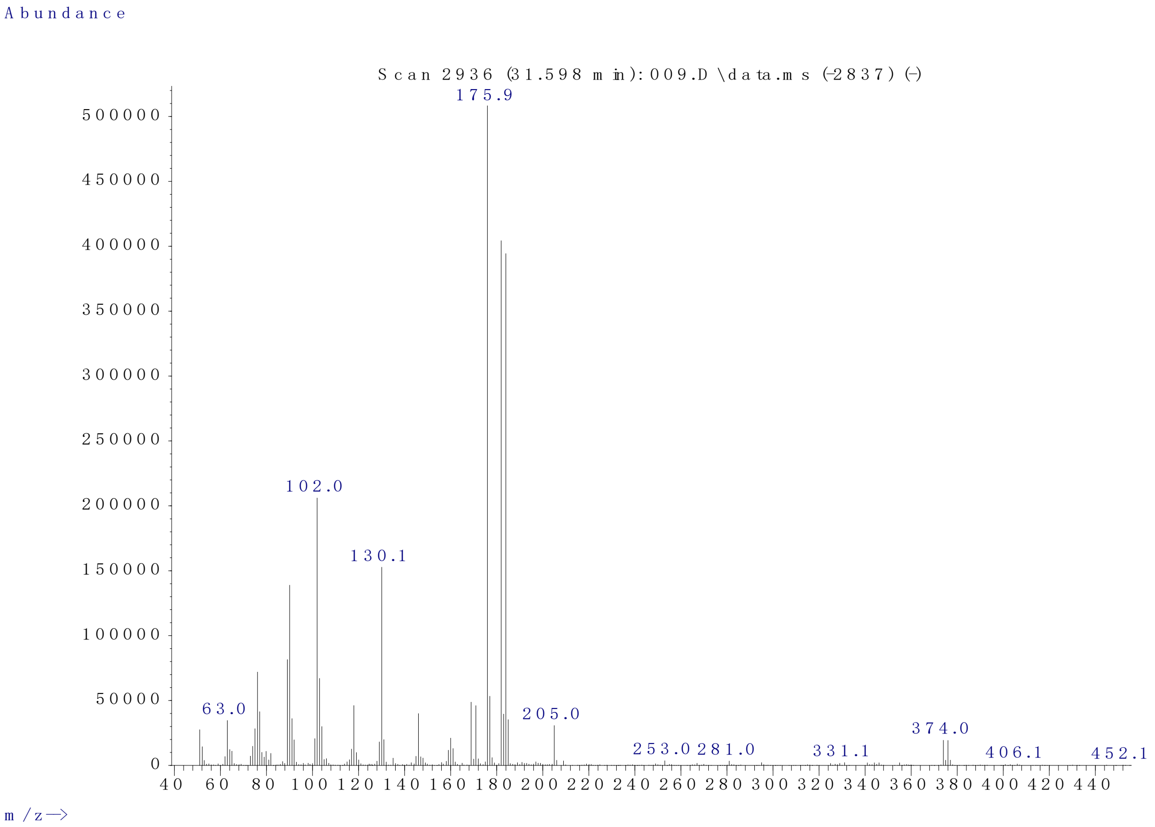


**Figure S35.** Mass spectrum of compound **6** ($CH_2Cl_2$).

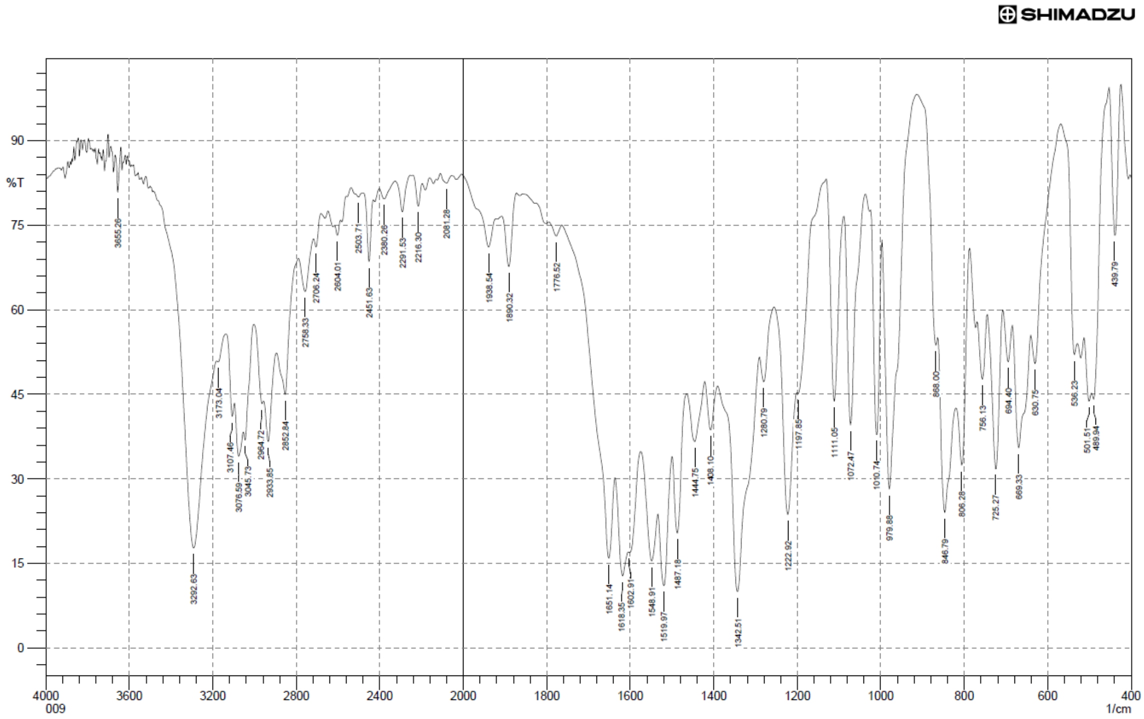


**Figure S36.** IR spectrum (KBr) of compound **6**.

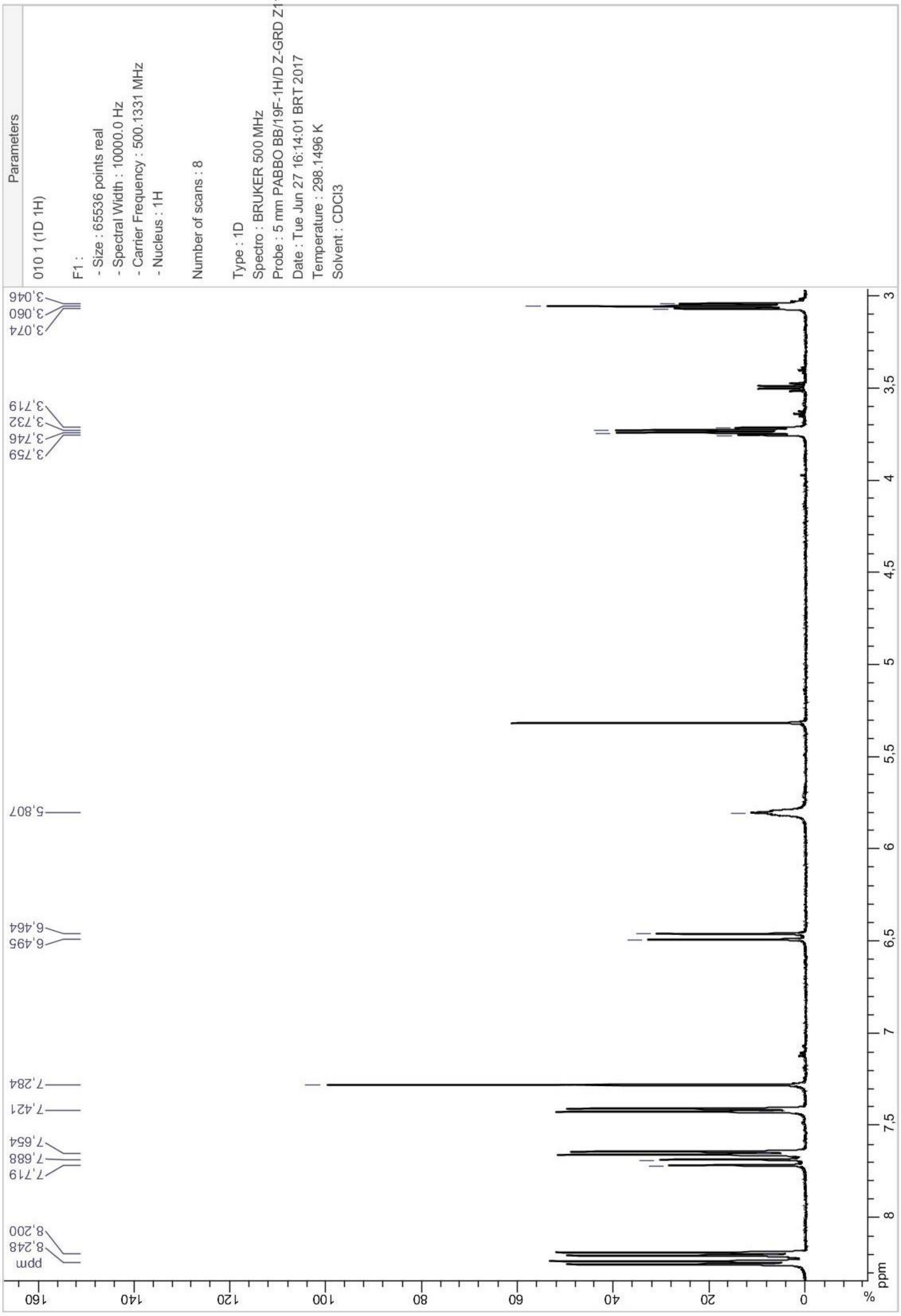


**Figure S37.** $^1$H NMR spectrum of compound **7** ($CDCl_3$, 500 MHz).

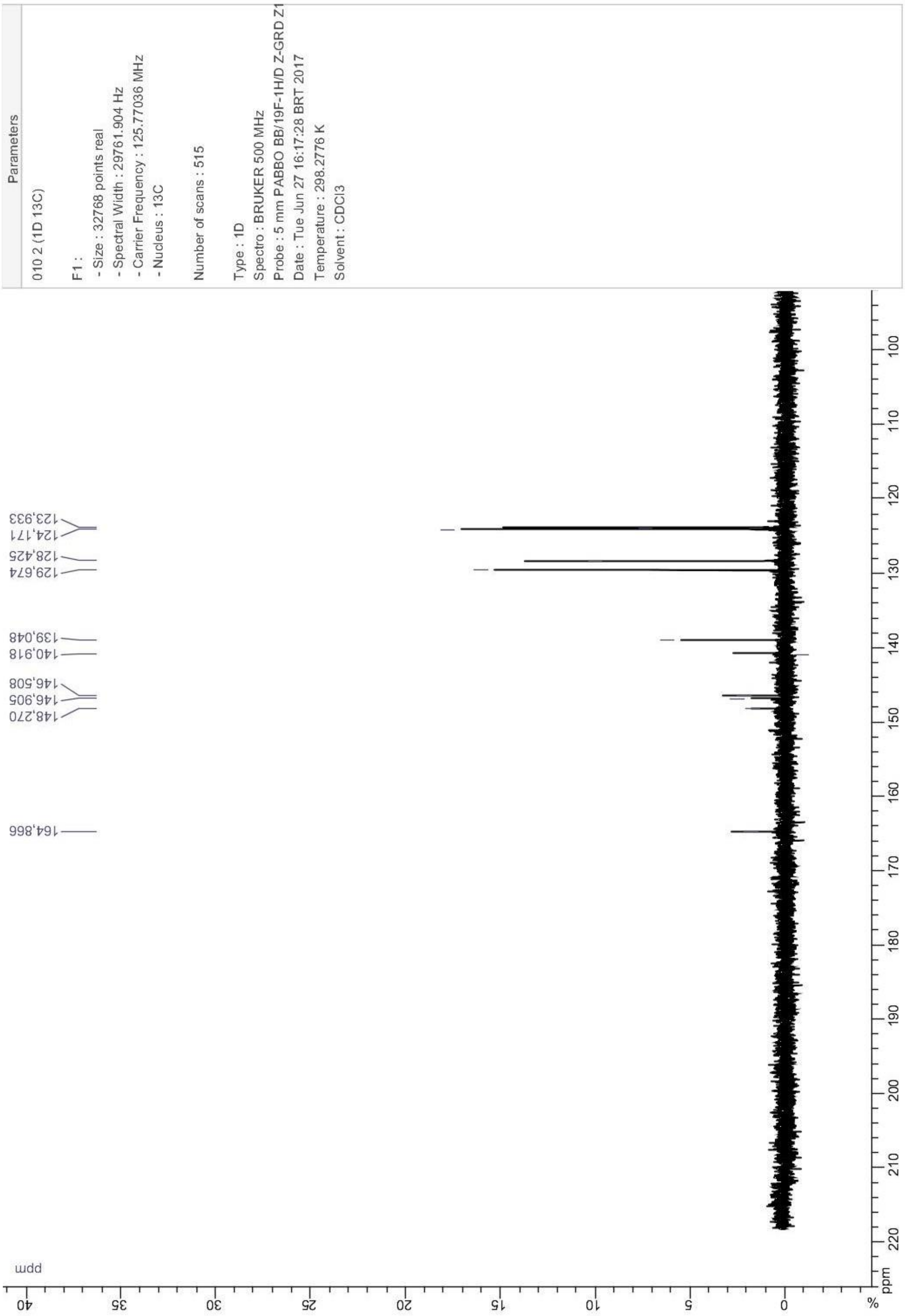


**Figure S38.** $^{13}C$ NMR spectrum of compound **7** ($CDCl_3$, 125 MHz).

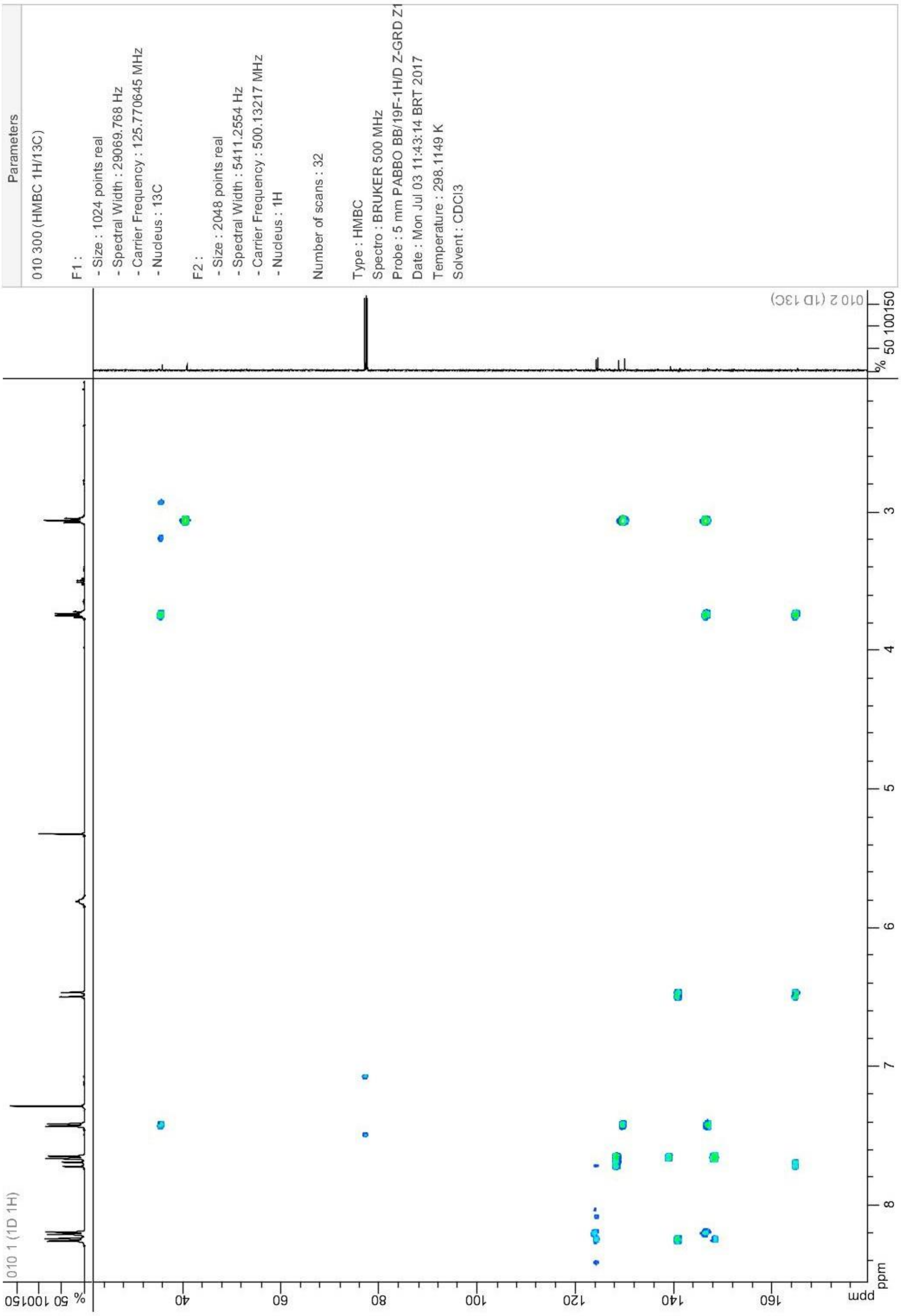


**Figure S39.** HMBC spectrum of compound **7** ($CDCl_3$, 500 MHz).

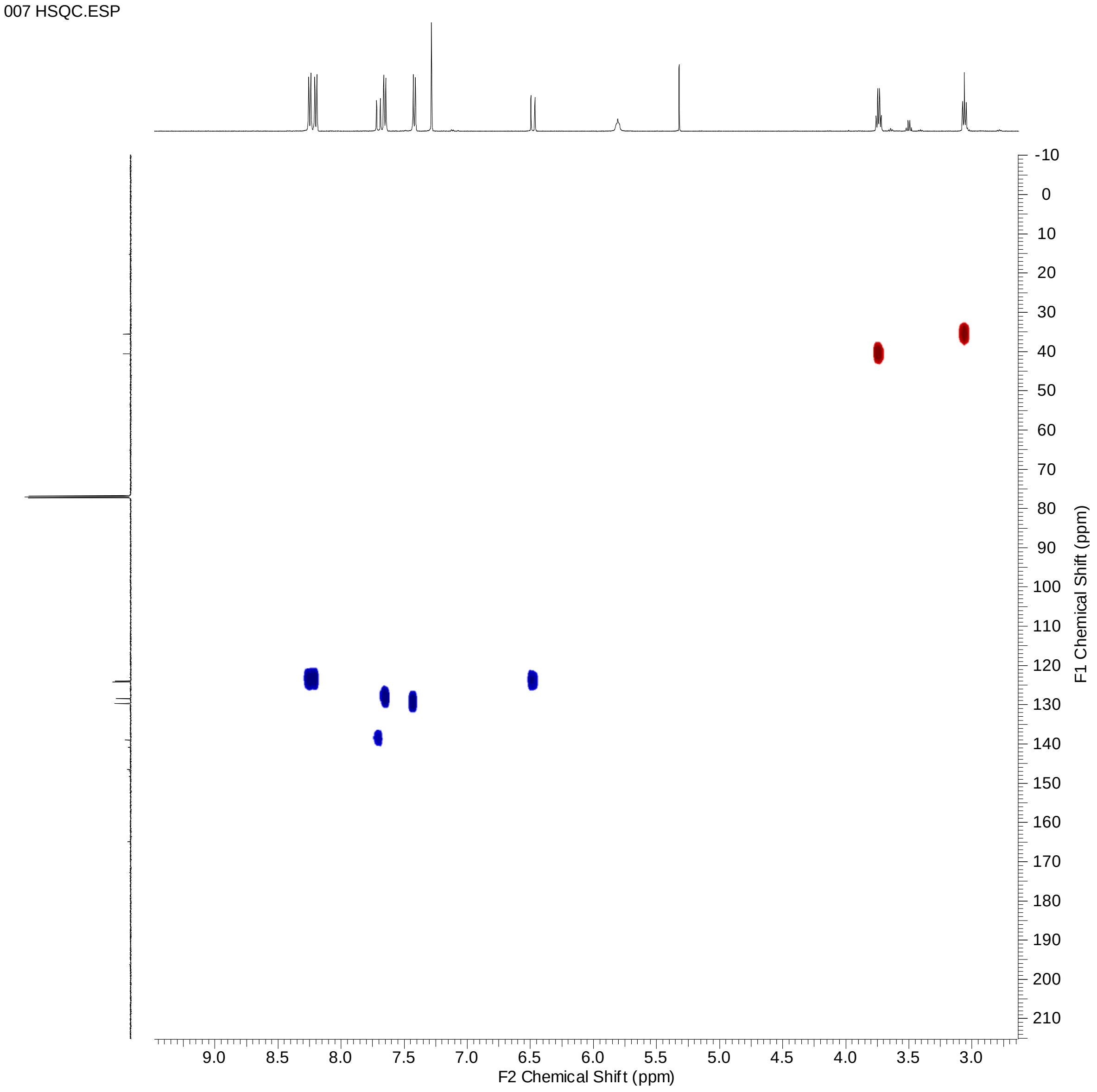


**Figure S40.** HSQC spectrum of compound **7** ($CDCl_3$, 500 MHz).

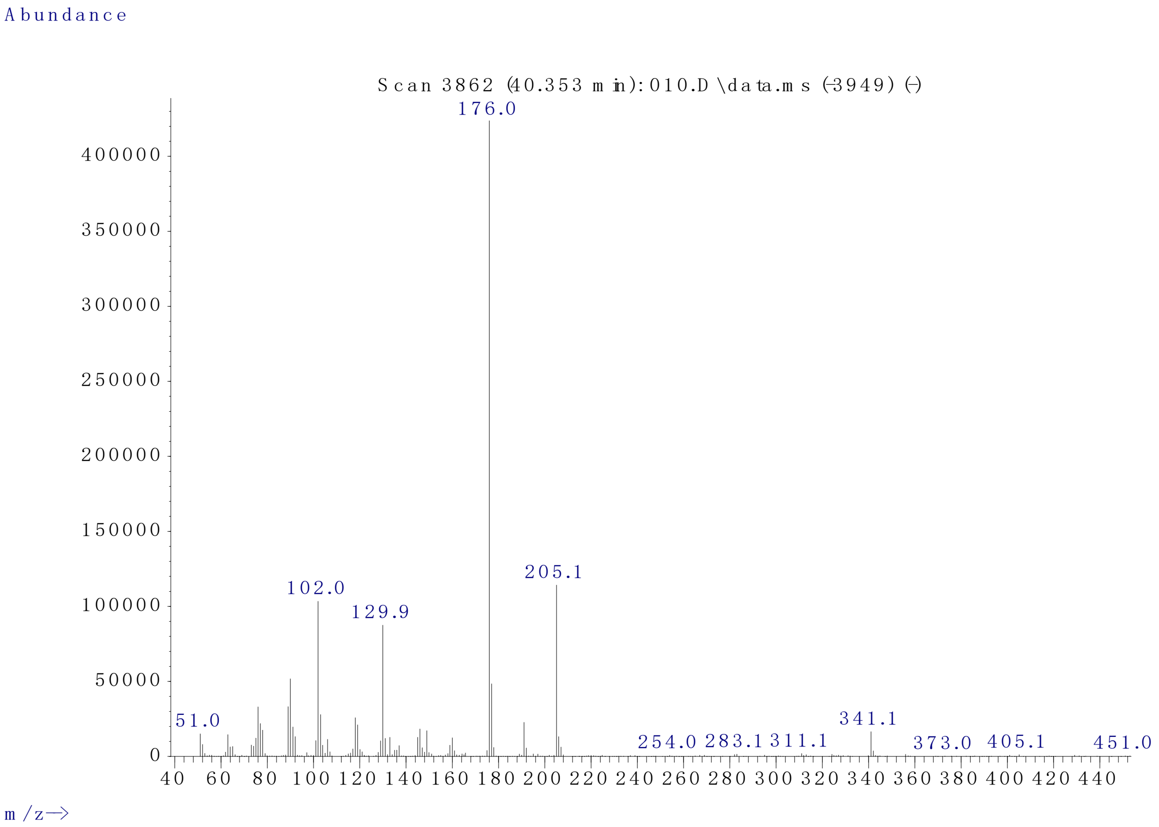


**Figure S41.** Mass spectrum of compound **7** ($CH_2Cl_2$).

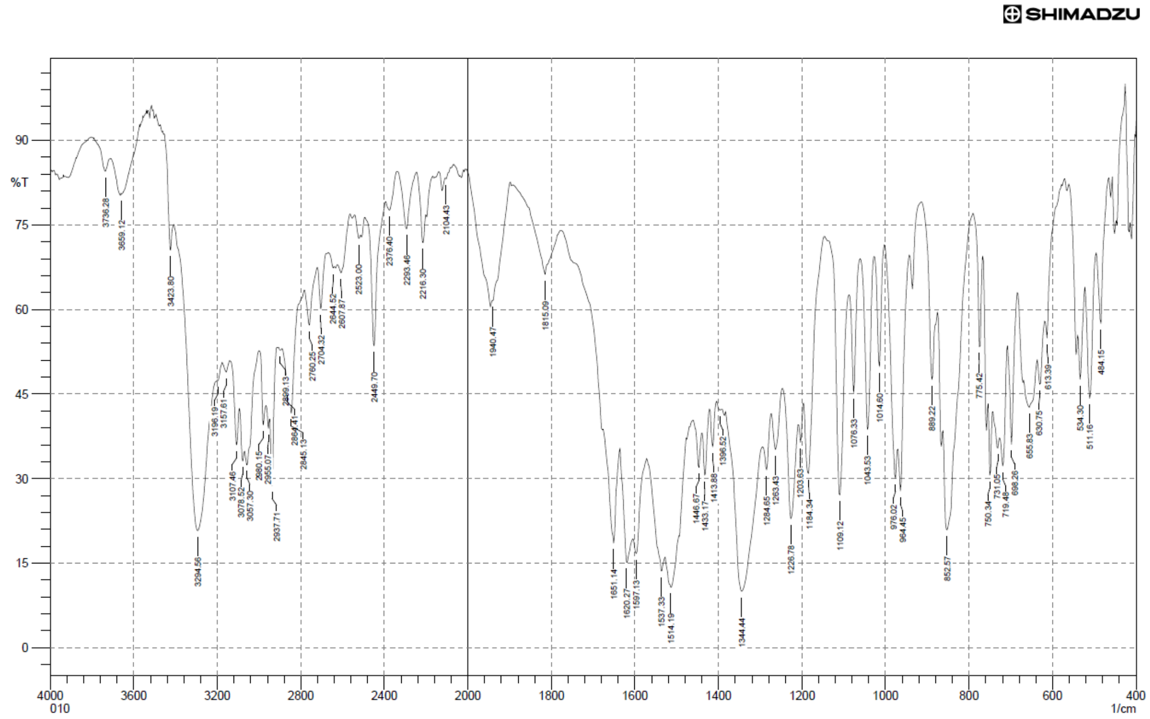

**Figure S42.** IR spectrum (KBr) of compound **7**.

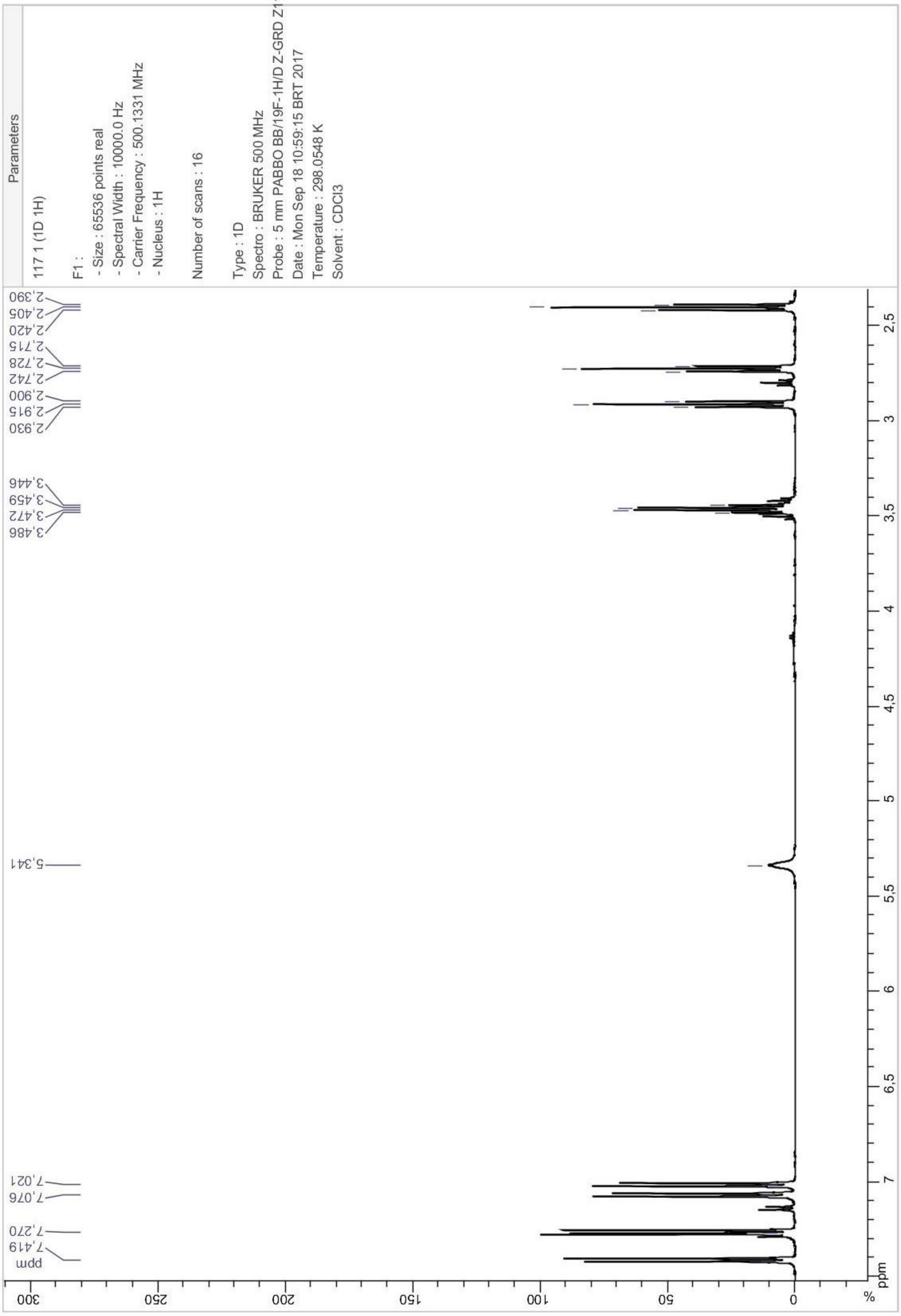


**Figure S43.** $^1$H NMR spectrum of compound **8** ($CDCl_3$, 500 MHz).

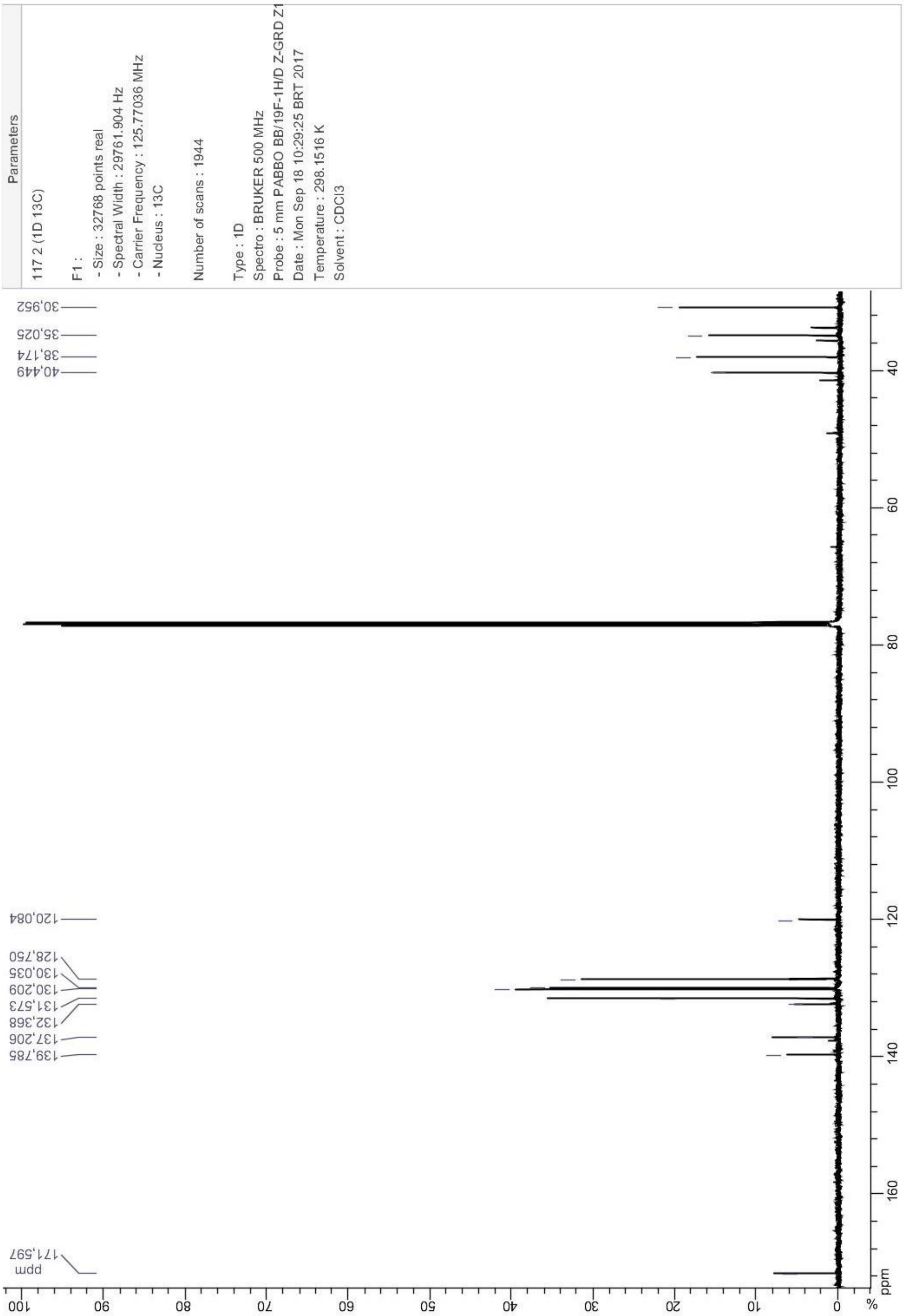


**Figure S44.** $^{13}C$ NMR spectrum of compound **8** ($CDCl_3$, 125 MHz).

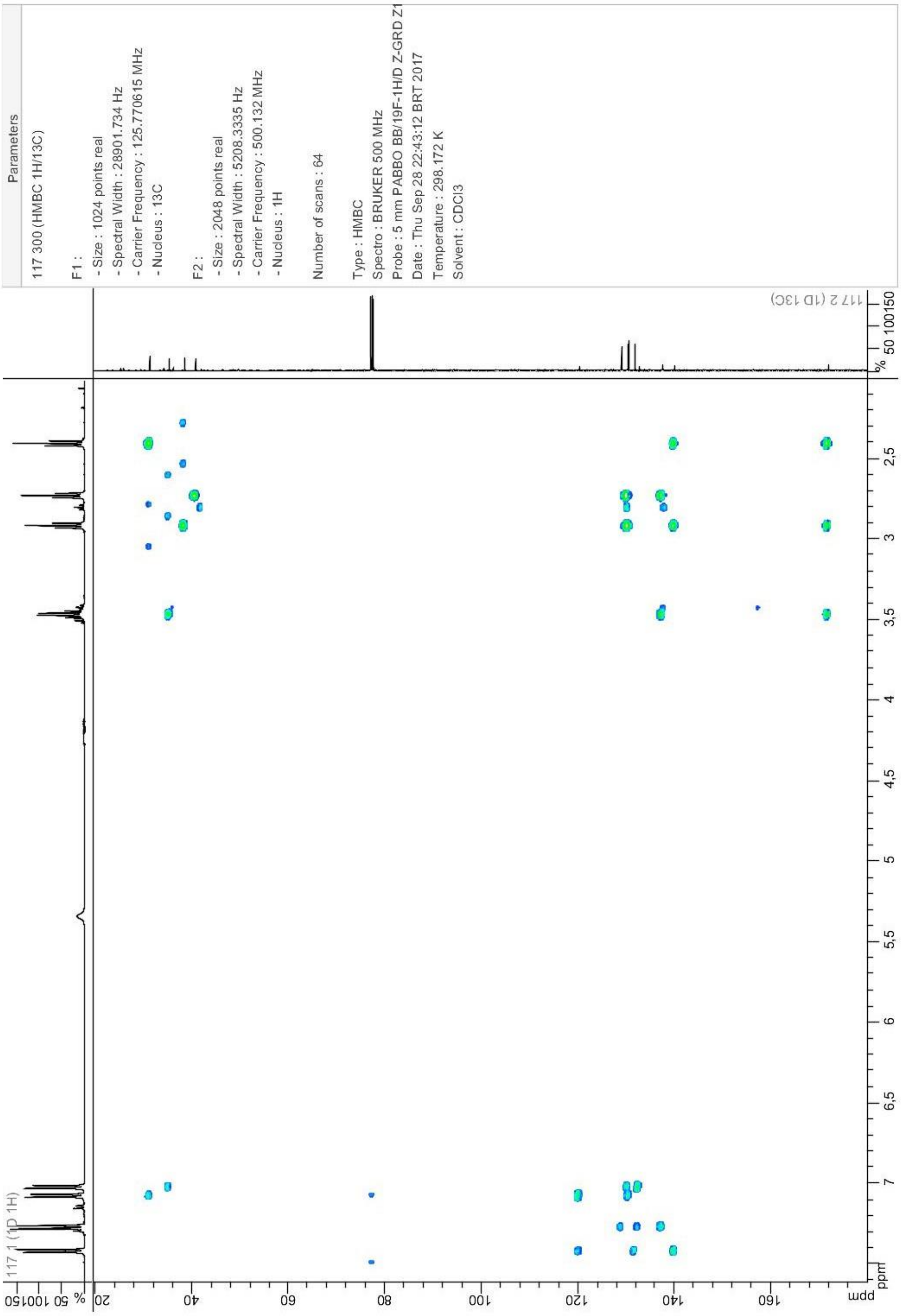


**Figure S45.** HMBC spectrum of compound **8** ($CDCl_3$, 500 MHz).

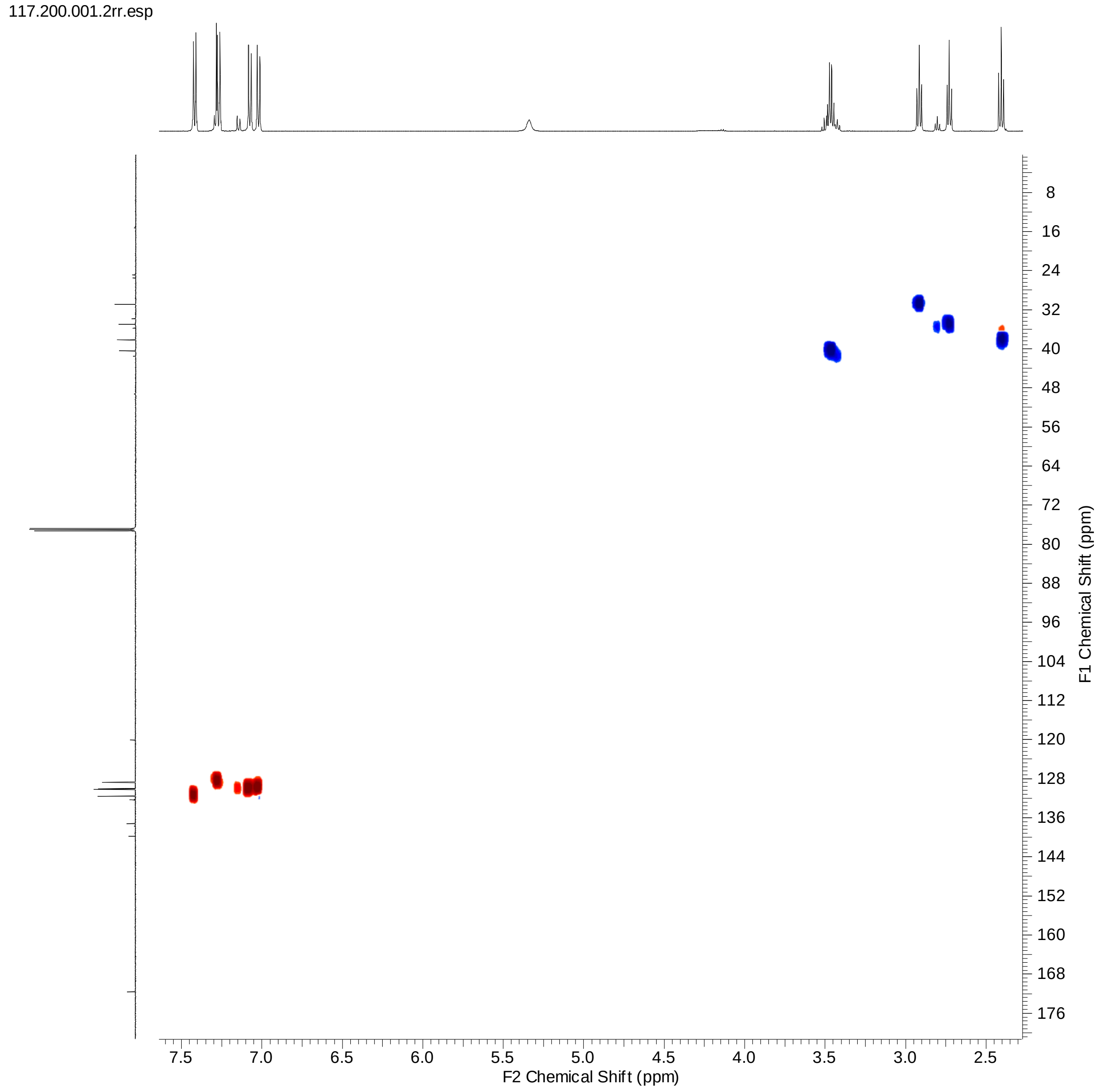


**Figure S46.** HSQC spectrum of compound **8** ($CDCl_3$, 500 MHz).

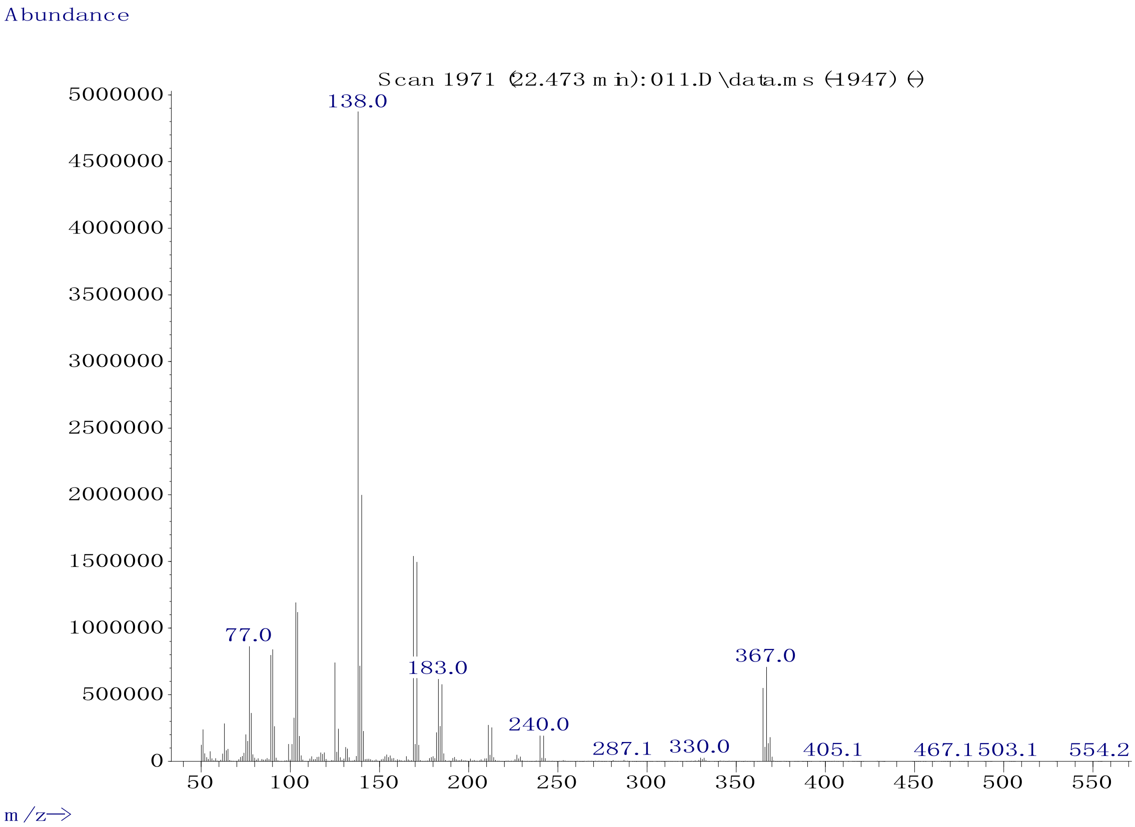


**Figure S47.** Mass spectrum of compound **8** ($CH_2Cl_2$).

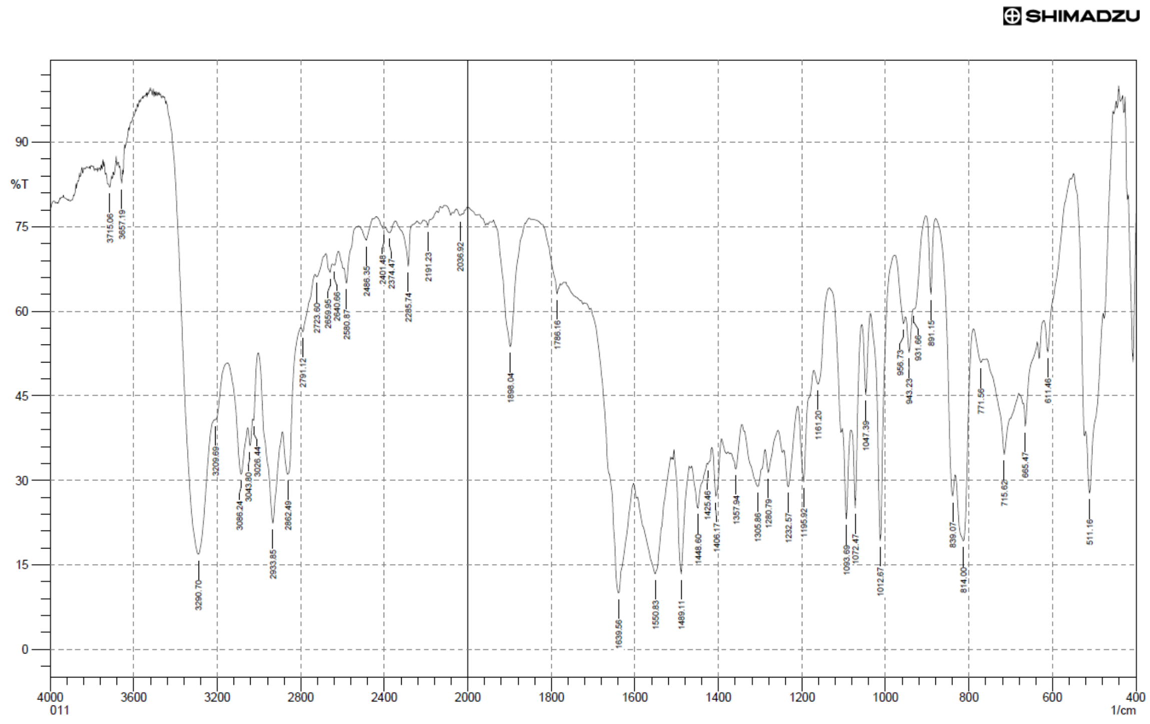


**Figure S48.** IR spectrum (KBr) of compound **8**.
